# Human adiponectin receptor AdipoR1 assumes closed and open structures

**DOI:** 10.1038/s42003-020-01160-4

**Published:** 2020-08-14

**Authors:** Hiroaki Tanabe, Yoshifumi Fujii, Miki Okada-Iwabu, Masato Iwabu, Kuniyuki Kano, Hiroki Kawana, Masakatsu Hato, Yoshihiro Nakamura, Takaho Terada, Tomomi Kimura-Someya, Mikako Shirouzu, Yoshiaki Kawano, Masaki Yamamoto, Junken Aoki, Toshimasa Yamauchi, Takashi Kadowaki, Shigeyuki Yokoyama

**Affiliations:** 1 RIKEN Structural Biology Laboratory, 1-7-22 Suehiro-cho, Tsurumi-ku, Yokohama 230-0045 Japan; 2 RIKEN Cluster for Science, Technology and Innovation Hub, 1-7-22 Suehiro-cho, Tsurumi-ku, Yokohama 230-0045 Japan; 3 Division of Structural and Synthetic Biology, RIKEN Center for Life Science Technologies, 1-7-22 Suehiro-cho, Tsurumi-ku, Yokohama 230-0045 Japan; 4 Department of Diabetes and Metabolic Diseases, Graduate School of Medicine, The University of Tokyo, 7-3-1 Hongo, Bunkyo-ku, Tokyo 113-0033 Japan; 5 Laboratory for Advanced Research on Pathophysiology of Metabolic Diseases, The University of Tokyo, 7-3-1 Hongo, Bunkyo-ku, Tokyo 113-0033 Japan; 6 Department of Integrated Molecular Sciences on Metabolic Diseases, 22nd Century Medical and Research Center, The University of Tokyo, 7-3-1 Hongo, Bunkyo-ku, Tokyo 113-0033 Japan; 7 JST, PRESTO, Honcho, Kawaguchi, Saitama 332-0012 Japan; 8 Laboratory of Molecular and Cellular Biochemistry, Graduate School of Pharmaceutical Sciences, Tohoku University, Miyagi, 980-8578 Japan; 9 AMED-CREST, Japan Agency for Medical Research and Development, Otemachi, Chiyoda-ku, Tokyo 100-0004 Japan; 10 Laboratory for Protein Functional and Structural Biology, RIKEN Center for Biosystems Dynamics Research, 1-7-22 Suehiro-cho, Tsurumi-ku, Yokohama 230-0045 Japan; 11 RIKEN SPring-8 Center, Kouto, Sayo, Hyogo 679-5148 Japan; 12 Department of Metabolism and Nutrition, Mizonokuchi Hospital, Faculty of Medicine, Teikyo University, 5-1-1 Futago, Takatsu-ku, Kawasaki, Kanagawa 213-8507 Japan

**Keywords:** Membrane proteins, X-ray crystallography

## Abstract

The human adiponectin receptors, AdipoR1 and AdipoR2, are key anti-diabetic molecules. We previously reported the crystal structures of human AdipoR1 and AdipoR2, revealing that their seven transmembrane helices form an internal closed cavity (the closed form). In this study, we determined the crystal structure of the D208A variant AdipoR1, which is fully active with respect to the major downstream signaling. Among the three molecules in the asymmetric unit, two assume the closed form, and the other adopts the open form with large openings in the internal cavity. Between the closed- and open-form structures, helices IV and V are tilted with their intracellular ends shifted by about 4 and 11 Å, respectively. Furthermore, we reanalyzed our previous wild-type AdipoR1 diffraction data, and determined a 44:56 mixture of the closed and open forms, respectively. Thus, we have clarified the closed-open interconversion of AdipoR1, which may be relevant to its functional mechanism(s).

## Abstract (web summary)

Tanabe, Fujii, Okada-Iwabu, Iwabu et al. report the crystal structures of the D208A variant of human adiponectin receptor AdipoR1 as well as the revised structures of the wild-type AdipoR1. These structures clarify the interconversion between the open and closed states of this protein, which may be relevant to its function.

## Introduction

Human AdipoR1 and AdipoR2 belong to the progestin and adipoQ receptor family^1^, and serve as the receptors for the antidiabetic adipokine, adiponectin. AdipoR1 activates the AMP-activated protein kinase (AMPK)^2–4^ pathways, while AdipoR2 activates the peroxisome proliferator-activated receptor-α (PPAR-α)^5,6^ pathways leading to the increased expression of uncoupling protein 2 (UCP2)^7^. Thereby, they regulate glucose and lipid metabolism, inflammation, and oxidative stress in vivo. Plasma adiponectin levels are reduced in obesity and type 2 diabetes, while the activation of AdipoR by the small-molecule AdipoR agonist AdipoRon was shown to ameliorate diabetes and increase exercise endurance, and concurrently prolong the shortened lifespan due to obesity^8^.

Human AdipoR1 and AdipoR2 contain seven-transmembrane (7TM) domains, with an internal N terminus and an external C terminus, which is the opposite configuration to that of G protein-coupled receptors^9,10^. We previously reported the crystal structures of human AdipoR1 and AdipoR2^11,12^. These structures revealed that the 7TM helices surround a large internal cavity, designated hereafter as the major cavity. In the major cavity, a zinc ion is coordinated by three His residues. This zinc-coordinated structure is reminiscent of the catalytic sites of hydrolytic enzymes. A conserved Asp residue exists near the zinc ion (Asp208 in AdipoR1 and Asp219 in AdipoR2). The D208A mutant of AdipoR1 [residues 89–375, designated hereafter as AdipoR1(A208)] retained the full ability to activate AMPK, whereas the corresponding D219A mutant of AdipoR2 [residues 100–386, designated as AdipoR2(A219)] lacked the ability to increase the expression of UCP2^12^. These findings suggested that the putative enzymatic activity is important for the AdipoR2 activity. In fact, there is an extra electron density near the zinc ion in the major cavity of AdipoR2 [Protein Data Bank (PDB) ID 3WXW], which might be the substrate or product of the putative hydrolytic activity of AdipoR2. However, the major cavity in the AdipoR2 structure is essentially closed, with only tiny openings^12^.

Recently, Vasiliauskaité-Brooks et al.^13^ reported structures of AdipoR2 that are quite similar to our previous closed-form structure, and interpreted the extra electron density in the major cavity as the free fatty acid most abundant in insect cells, oleic acid. Accordingly, they suggested that the free fatty acid is the product of the putative ceramidase activity of AdipoR2^13^. In addition, they reanalyzed our previous data set for AdipoR1, and proposed that AdipoR1 assumes the open form (PDB ID 5LXG)^13^, rather than the closed form that we reported^12^.

In this study, we determined the crystal structure of the above-mentioned AdipoR1(A208) at 3.1 Å resolution. The asymmetric unit contains three molecules: two assume the closed form, and the other assumes the open form. Furthermore, we reanalyzed our previous wild-type AdipoR1 diffraction data [residues 89–375, designated hereafter as AdipoR1(D208)] using the closed and open structures of AdipoR1(A208), and refined the structure as a dual conformation consisting of the closed and open forms (44:56). Therefore, AdipoR1 adopts both the closed and open forms, probably in equilibrium, and their interconversion may be related to the signaling mechanism.

## Results

### Crystal structure of AdipoR1(A208)

**Fig. 1:**
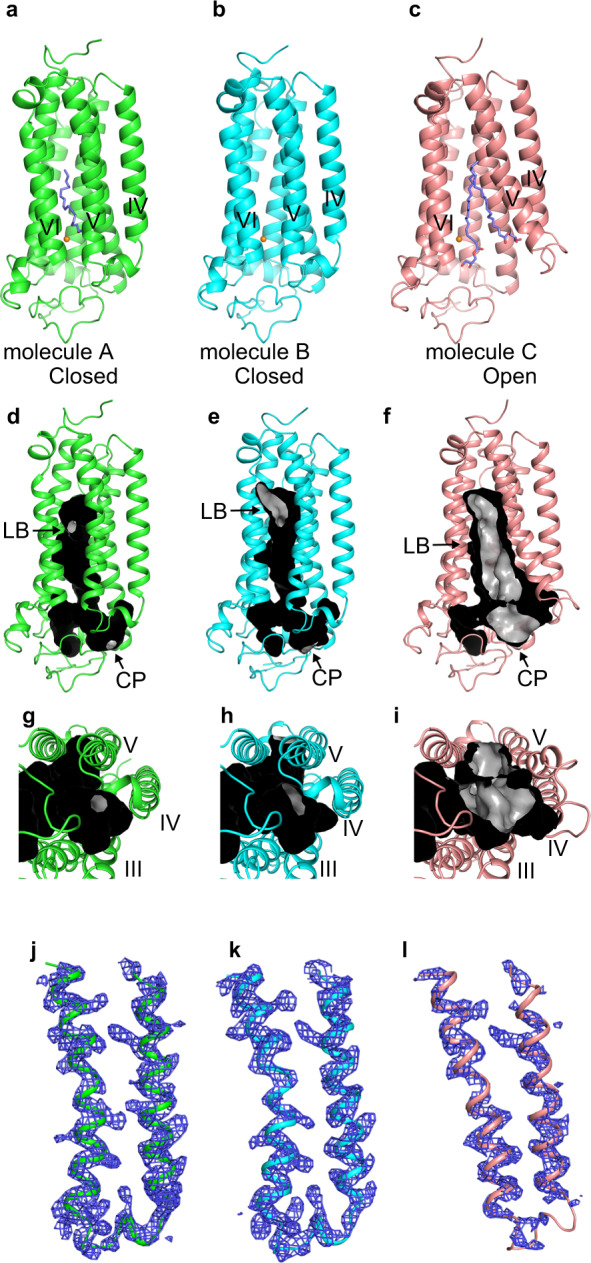
Crystal structure of human AdipoR1(A208). The crystal structure of human AdipoR1(A208) at 3.1-Å resolution: the asymmetric unit contains three molecules, A, B, and C (**a**–**c**). Molecules A (green) (**a**) and B (cyan) (**b**) are in the closed form, and molecule C (salmon) (**c**) is in the open form. The structures are viewed parallel to the membrane. The zinc ion is shown as an orange sphere. Two oleic acid molecules in molecule A and two monoolein molecules in molecule C are shown as slate blue stick models. **d**–**i** The major cavities of molecules A (**d**, **g**, green) and B (**e**, **h**, cyan) in the closed form, and molecule C (**f**, **i**, salmon) in the open form of AdipoR1(A208). The outside and inside surfaces of the major cavities are colored black and gray, respectively. The major cavities are viewed parallel to the membrane (**d**–**f**) and from the intracellular side (**g**–**i**). The lipid-bilayer (LB) and cytoplasmic (CP) openings are labeled LB and CP, respectively. Other minor cavities are omitted for clarity. **j**–**l** The simulated-annealing *F*_o_–*F*_c_ omit maps, contoured at 2.5*σ*. The electron densities on helices IV and V and ICL2 of molecules A (**j**, green) and B (**k**, cyan) in the closed form, and molecule C (**l**, salmon) in the open form of AdipoR1(A208). For comparison, molecules C and A are shown in gray in **j**/**k** and **l**, respectively.

We expressed AdipoR1(A208) in FreeStyle 293-F cells, purified it as a complex with the F_v_ fragment of a monoclonal antibody^12^, and crystallized the complex by the lipidic mesophase method^14,15^ with monoolein. By molecular replacement using our previous AdipoR1(D208) structure (3WXV)^12^ as the search model, we determined the crystal structure of AdipoR1(A208) at 3.1 Å resolution (PDB ID 6KRZ). The final refinement statistics of the AdipoR1(A208) crystal structure are shown in Tables 1 and 2. The asymmetric unit contains three independent molecules, A, B, and C (Fig. 1a–c). The packing of the three molecules in the crystal is shown in Supplementary Fig. 1. The root-mean-square deviation (r.m.s.d.) values for the main-chain Cα atoms are 0.701 Å between molecules A and B, 1.030 Å between molecules B and C, and 1.402 Å between molecules C and A.

**Table 1:** X-ray data collection and refinement statistics.

	AdipoR1(A208) PDB ID 6KRZ	AdipoR1(D208) PDB ID 6KS0^b^	AdipoR2(D219) PDB ID 6KS1^b^
Data collection			
No. of crystals	1	5	1
X-ray source	BL32XU, SPring-8	BL32XU, SPring-8	BL32XU, SPring-8
Wavelength (Å)	1	1	1
Space group	*P*2_1_2_1_2_1_	*C*222_1_	*P*2_1_2_1_2
Cell dimensions			
* a*, *b*, *c* (Å)	104.589 119.35 197.814	92.663, 194.443, 74.384	74.58, 108.63, 101.03
α, *β*, *γ* (°)	90.0, 90, 90.0	90.0, 90.0, 90.0	90.0, 90.0, 90.0
Resolution (Å)^a^	46.55–3.048 (3.157–3.048)	19.94–2.785 (2.884–2.785)	19.52–2.4 (2.486–2.4)
* I*/σ(*I*)^a^	7.33 (1.27)	7.70 (1.63)	8.55 (1.19)
* CC*_1/2_^a^	0.994 (0.478)	0.992 (0.439)	0.996 (0.504)
Completeness (%)^a^	99.72 (98.12)	95.67 (87.18)	97.48 (94.85)
Redundancy^a^	2.0 (2.0)	8.2 (7.7)	4.5 (4.5)
Refinement			
Resolution (Å)^a^	46.55–3.048 (3.157–3.048)	19.94–2.785 (2.884–2.785)	19.52–2.4 (2.486–2.4)
No. of reflections^a^	47,908 (4645)	16,498 (1458)	32,174 (3200)
* R*_work_/*R*_free_^c^	0.2159/0.2726	0.2209/0.2773^e^	0.2288/0.2673^f^
* R*^xpct^_work_/*R*^xpct^_free_^d^		0.198/0.269^e^	0.185/0.239^f^
No. of atoms			
Protein	12,111	4297	4081
Ligands	101	6	201
Water	89	30	201
* B*-factors			
Protein	74.79	78.57	82.85
Ligands	101.41	55.98	105.99
Water	43.28	58.90	67.59
R.m.s. deviations			
Bond lengths (Å)	0.003	0.002	0.004
Bond angles (°)	0.44	0.51	0.58

^a^ Values in parentheses are for highest-resolution shell.

^b^ The PDB entries 6KS0 and 6KS1 have replaced 3WXV and 3WXW, respectively.

^c^ The final *R*_work_/*R*_free_ values were obtained with phenix.refine (see the “Methods” section).

^d^ The final *R*^xpct^_work_/*R*^xpct^_free_ values were obtained with autoBuster (see the “Methods” section).

^e^ For comparison with these values, the final *R*_work_/*R*_free_ and *R*^xpct^_work_/*R*^xpct^_free_ values for the previous single open-form structure (PDB ID 5LXG)^13^ were estimated to be 0.2357/0.2799 and 0.223/0.267, respectively (see the “Methods” section).

^f^ For comparison with these values, the final *R*_work_/*R*_free_ and *R*^xpct^_work_/*R*^xpct^_free_ values for the previously revised structure (PDB ID 5LWY)^13^ were estimated to be 0.2224/0.2642 and 0.191/0.231, respectively (see the “Methods” section).

**Table 2:** Residues of molecule A of AdipoR1(A208) interacting with the oleic acid molecules OLA1 and OLA2 in the crystal.

Molecule A					
OLA1			OLA2		
Y	209	III	S	219	III
L	215	III	F	220	III
S	219	III	G	275	V
A	268	V	L	276	V
F	271	V	G	278	V
L	272	V	W	302	VI
M	306	VI	F	303	VI
Y	310	VI	M	306	VI
A	314	VI	A	307	VI
Y	317	VI	Y	310	VI
F	340	VII	H	351	VII
V	344	VII			
A	347	VII			
A	348	VII			
H	351	VII

### The overall structures of the closed and open forms of AdipoR1(A208)

Molecules A, B, and C of AdipoR1(A208) exhibit the 7TM helices that form the major cavity (Fig. 1d–f). The zinc-coordination structure of the putative hydrolytic catalytic site is conserved in the three molecules (Supplementary Fig. 2). Molecules A and B assume the closed form (Fig. 1a, b) and molecule C assumes the open form (Fig. 1c).

In the closed-form structures of molecules A and B, the major cavity is mostly closed, with only two small openings, one to the outer leaflet of the lipid bilayer [lipid-bilayer (LB) opening] and the other to the cytoplasm [cytoplasmic (CP) opening] (Fig. 1d, e, g, h). These closed-form structures of AdipoR1(A208) are similar to our previous closed-form structure of AdipoR1(D208) and to those of AdipoR2(D219)^12,13^. The closed cavity of molecule A contains two oleic acid molecules (Fig. 1a, Supplementary Notes). The LB opening is smaller in molecule A than in molecule B (Fig. 1d, e). The structure of molecule A of AdipoR1(A208) is hereafter described as the representative of the closed form, unless otherwise noted.

In contrast, in the open-form structure of molecule C, both the LB and CP openings of the major cavity are much larger than those in the closed-form structures of molecules A and B (Fig. 1f, i). Two monoolein molecules, the host lipid of the mesophase crystallization, are bound in the major cavity of molecule C (Fig. 1c).

**Fig. 2:**
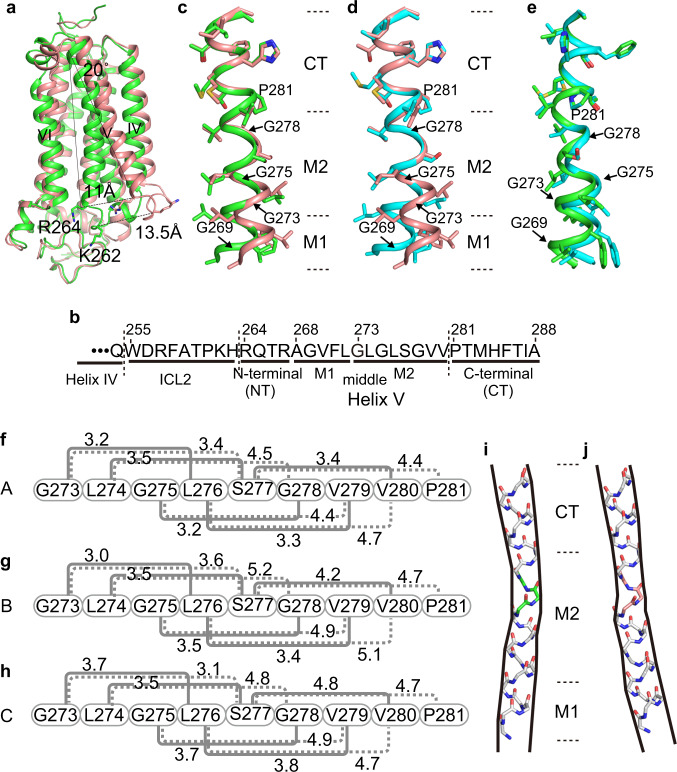
Structures of helix V in the closed and open forms of AdipoR1(A208). **a** Superimposition of the closed-form (molecule A, green) and open-form (molecule C, salmon) structures. The cytoplasmic ends of helices V are shifted by 11 Å between the two structures. **b** The amino acid sequences of ICL2 (Trp255–His263) and helix V (Arg264–Ala288), further divided into the N-terminal (NT) region (Arg264–Arg276), the middle (M1 and M2) regions (Ala268–Leu272 and G273–Val280, respectively), and the C-terminal (CT) region (Pro281–Ala288), of AdipoR1. **c**–**e** Structure comparison along the M1, M2, and CT regions of helix V among molecules A (green), B (cyan), and C (salmon), by superimposition of the CT region. **f**–**h** The distances (Å) of the main-chain amide carbonyl oxygen atom (CO) of residue *i* with the main-chain amide nitrogen atom (HN) of residues *i* + 3 (solid gray line) and *i* + 4 (dashed gray line) in the M2 region (*i* = 273–277) of helix V of molecules A (**f**), B (**g**), and C (**h**). According to these CO–HN distances and the bond angles in the M2 region (Supplementary Table 1), a 3_10_-helical conformation, rather than an α-helix, is assumed with four weak hydrogen bonds (3.2–3.5 Å) between the COs of Leu274–Ser277 (residues *i*) and the HNs of Ser277–Val280 (residues *i* + 3) in molecule A (**f**), whereas such 3_10_-helical hydrogen bonding is weaker in molecule B (**g**) and almost negligible in molecule C (**h**). The CO of Gly273 does not form a hydrogen bond in molecules A–C (Supplementary Table 1). **i**, **j** Schematic representations of the main chain of helix V, which is bent inward in molecule A (**i**) and rather straight in molecule C (**j**). The 3_10_-helical conformations of Leu276–Ser277–Gly278, colored green in molecule A (**i**) and salmon in molecule C (**j**), in the center of the M2 region are thinner than the α-helical conformations in the M1 and CT regions.

Helix V and intracellular loop 2 (ICL2), which connects helices IV and V, adopt different structures between the closed and open forms (Fig.1j–l). Typically, helix V is differentially bent, and its N-terminal end (Arg264) is shifted outward by about 11 Å between molecules A and C (Fig. 2a, Supplementary Fig. 3a). Both helix V and ICL2 are substantially shifted: Lys262 within ICL2 is repositioned by as much as 13.5 Å (Fig. 2a, Supplementary Fig. 3a). In addition, helix IV is concomitantly tilted, and its C-terminal, CP end (Gln254) is shifted by 3.6 Å between the two forms (Supplementary Fig. 3a).

### The M2 region of helix V is highly pliable due to the Gly and Pro residues in AdipoR1(A208)

The conformational properties of helix V vary along its sequence, the N-terminal (NT) region (Arg264–Arg276), the middle regions M1 (Ala268–Leu272) and M2 (Gly273–Val280), and the C-terminal (CT) region (Pro281–Ala288) (Fig. 2b). First of all, the CT region is rigidly fixed to the main body of AdipoR1, by the interactions of Pro281–Thr286 with a number of residues from helices III, IV, and VI (Supplementary Fig. 4). The helix axis of the CT region of helix V is tilted by ~20°, as compared to those of helices IV and VI (Fig. 2a). Two residues, Ile287 and Ala288, at the end of helix V are exposed on the extracellular surface of AdipoR1.

The conserved Pro residue, Pro281, at the beginning of the CT region, and four Gly residues, at positions 269 in the M1 region and 273, 275, and 278 in the M2 region (Fig. 2b), are the key residues for the bent structures of helix V. The M1 region assumes the standard α-helix. On the other hand, when the CT region is superimposed between molecules A–C, the M2 region is bent at Gly278, Gly275, and/or Gly273 in different manners (Fig. 2c–e).

The bent structures of the M2 region are relevant to the uncommon structural property initiated by the imino-acid residue Pro281, which specifically lacks the hydrogen at the main-chain nitrogen atom. The main-chain carbonyl oxygen atom of its fourth upstream residue Ser277 (Ser277-CO) lacks a hydrogen-bonding partner, and is located 4.4–4.7 Å away from the main-chain nitrogen atom of Pro281, due to its steric repulsion with the δ-methylene group of the proline side chain (CN-Pro281) (Fig. 2f–h, Supplementary Fig. 5a–c). The conformational irregularity is propagated from both sides of Ser277 in the M2 region (Fig. 2b), probably because of Gly278, Gly275, and Gly273, resulting in an unusual conformation with a type I *β*-turn or 3_10_-helix, according to the DSSP program^16^. The conformational details are as follows.

On the N-terminal side of Ser277, the main-chain HN group (HN-Ser277) (*j*) cannot hydrogen bond with the main-chain CO group of Gly273 (Gly273-CO) (*j*–4), as judged from the O···N distance (3.4–3.6 Å) and/or the O···H−N angle (102–125°) (Supplementary Table 1)^17,18^, in molecules A–C. Instead, HN-Ser277 (residue *j*) may hydrogen bond with Leu274-CO (residue *j*–3) (Fig. 2f–h, Supplementary Table 1). Similarly, the main-chain O···N distances are shorter between residues *i* and *i* + 3 than between residues *i* and *i* + 4 by 0.9–1.7 Å for *i* = 274–276, in molecules A–C. Thus, on both sides of Ser277, this 3_10_-helical conformation is formed over the seven residues Leu274–Val280. In molecule A, the O···N distances and the O···H−N angles between the residues from Leu274-CO to Ser277-CO and those from HN-Ser277 to HN-Val280 are 3.2–3.5 and 145–164° (Supplementary Table 1), respectively, indicating that four weak hydrogen bonds stabilize the 3_10_-helix. In molecule B, three weak hydrogen bonds are formed: the O···N distances and the O···H−N angles between the three residues from Leu274-CO to Leu276-CO and those from HN-Ser277 to HN-Val279 are 3.4–3.5 Å and 159–171° (Supplementary Table 1), respectively. On the other hand, only one weak hydrogen bond is formed between Leu274-CO and HN-Ser277 in molecule C. Therefore, the 3_10_-helical conformation of Leu274–Val280 is pliable, as it is considerably less stabilized by hydrogen bonding than a regular α-helical conformation.

The (ϕ, ψ) angles of Leu276, Ser277, and Gly278 are more similar to those in a 3_10_-helix than an α-helix (Supplementary Table 2). Thus, the helix formed by Leu276–Ser277–Gly278 is appreciably narrower (~3 residues per turn) than the standard α-helix (3.6 residues per turn) (Supplementary Fig. 6). Overall, the M2 region with weak hydrogen bonds assumes a stretched structure: residues Gly273–Val280 exhibit an advance of 11.4–12.5 Å (Supplementary Fig. 5d–f), which is appreciably longer than the advance of 10.5 Å in the standard α-helix.

At the N terminus of the M2 region, Gly273-CO (*i*) does not hydrogen bond with either HN-Leu276 (*i* + 3) or HN-Ser277 (*i* + 4), because the O H−N angles (102–125°) are smaller than 130°^17,18^ (Supplementary Table 1). Therefore, the 3_10_-helical conformation is terminated at Gly273-CO. On the other hand, HN-Gly273, HN-Leu274, HN-Gly275, and HN-Leu276 of the M2 region hydrogen bond with Gly269-CO, Val270-CO, Phe271-CO, and Leu272-CO, respectively, of the M1 region, as in the regular α-helix (Supplementary Table 1). In this manner, Gly273 serves as the junction between the 3_10_- and α-helical conformations. Similarly, on the other side of the M2 region, Gly278-CO, Val279-CO, and Val280-CO hydrogen bond with HN-Thr282, HN-Met283, and HN-His284 of the CT region. Consequently, the non-hydrogen-bonded Gly273-CO and CN-Pro281 delimit the 3_10_-helical conformation of Leu274–Val280, contributing further to the pliability of the M2 region.

Thus, the helical conformations in the M1, M2, and CT regions of helix V consist of an α-helix (M1), 3_10_-helix (M2), and α-helix (CT), and the two α-helices of the CT and M1 regions are not coaxial with each other (Fig. 2i, j, Supplementary Fig. 5g–i). For the M2 region, the degrees of bending are drastically different between the closed (molecules A and B) and open (molecule C) forms (Fig. 2c, d). Interestingly, the degrees of bending are appreciably distinct even between molecules A and B, which are both in the closed form (Fig. 2e).

### The M1 region of helix V is tied to helices III and IV in the open form of AdipoR1(A208)

In the closed form, helix V is bent inward by ~18° within the M2 region (Fig. 2i). In contrast, helix V assumes a rather straight orientation, in which the helix axis is nearly parallel between the M1 and CT regions in the open form (Fig. 2j). The M2 region itself only weakly interacts with other TM helices. Thus, the fixation of helix V, with the highly pliable M2 region adopting different structures between the closed and open forms (Fig. 2i, j), is ascribed to its different degrees of interactions on the N-terminal side with other parts of AdipoR1, as follows.

**Fig. 3:**
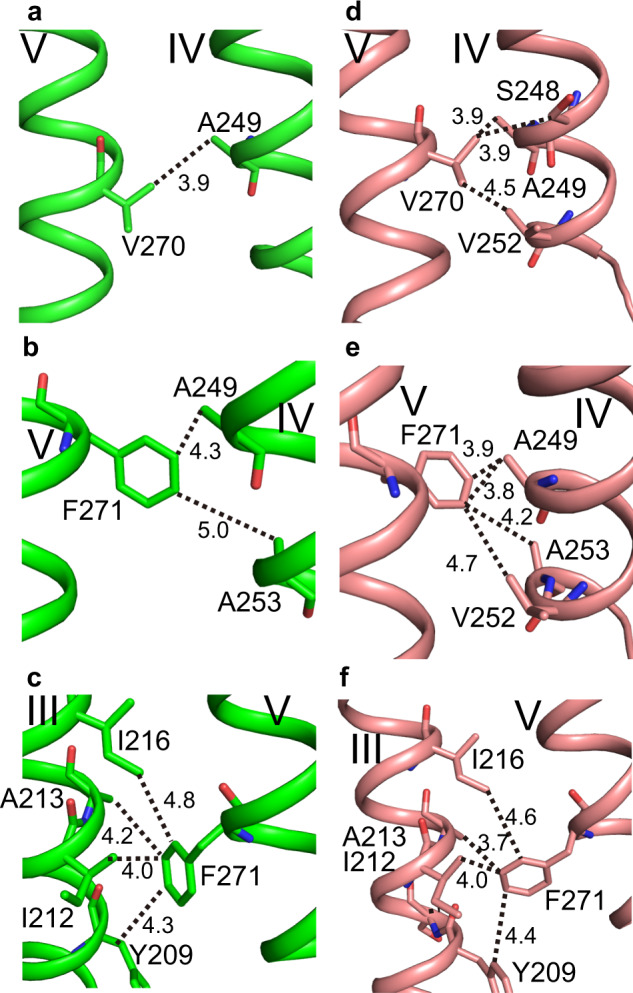
Interactions of the M1 region of helix V with helices III and IV in the closed and open forms of AdipoR1(A208). Hydrophobic interactions of helix V in the closed form (molecule A) (**a**–**c**) and the open form (molecule C) (**d**–**f**). Val270 of helix V interacts with helix IV (**a**, **d**), and Phe271 of helix V interacts with helix IV (**b**, **e**) and helix III (**c**, **f**). Overall, the degrees of the hydrophobic interactions are much more intensive in the open form (**d**–**f**) than the closed form (**a**–**c**).

The degrees of interactions of the M1 region of helix V with helices III and VI are shown for the closed form (Fig. 3a–c) and the open form (Fig. 3d–f). The open-form-specific structure of helix V is fixed mainly through the hydrophobic interactions of Val270 and Phe271 in the M1 region with helix IV (Fig. 3d–f). The methyl groups of Val270 interact with the methyl groups of Ala249 and Val252 and the methylene group of Ser248 (Fig. 3d). The phenyl ring of Phe271 interacts with the methyl groups of Ala249, V252, and Ala253 (Fig. 3e). These intensive hydrophobic interactions between helices IV and V in the open form (Fig. 3d, e) contrast sharply with the weak interactions in the closed form (Fig. 3a, b). Moreover, in the open form, Phe271 also makes hydrophobic interactions with Tyr209, Ile212, Ala213, and Ile216 from helix III (Fig. 3f), which are stronger than those in the closed form (Fig. 3c). Thus, helix V is immobilized firmly between helices III and IV in the open form, by extensive hydrophobic interactions. To bring Val270 and Phe271 of helix V close to helices III and IV, helix V assumes the straight orientation (Fig. 2j). These features of the M1 region of helix V maintain the large LB aperture in the open form.

In contrast, in the closed form, the M1 region of helix V forms only a few interactions and is almost detached from helix IV (Fig. 3a, b), and weakly interacts with helix III as compared with the open form (Fig. 3c). Nevertheless, helix V is fixed in the closed-form-specific inward-bent structure (Fig. 2i), and the M1 and M2 regions cover the outer-leaflet side of the LB opening (Fig. 1d, e, g, h). The fixation of helix V in the closed-form-specific bent structure and the concomitant minimization of the LB opening of the major cavity are ascribed to the interactions of the NT region of helix V and ICL2 with other parts of the protein, as described below.

### The NT region of helix V and ICL2 are tied to helices III and VI and the N-terminal globular domain in the closed form of AdipoR1(A208)

**Fig. 4:**
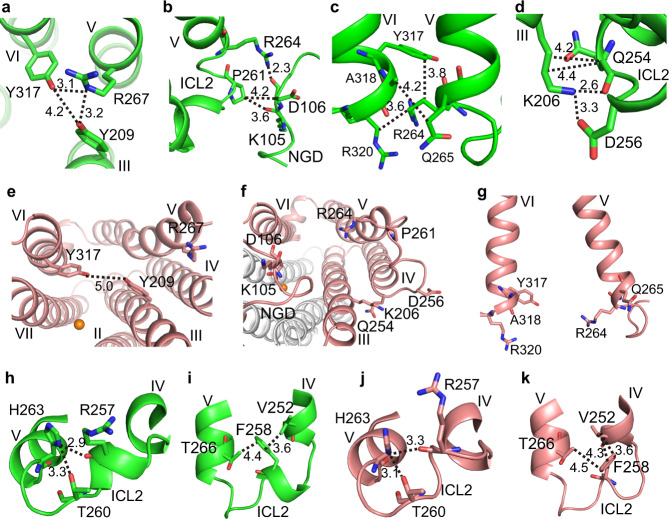
Local structures and interactions of the NT region of helix V and ICL2 in the closed and open forms of AdipoR1(A208). **a**–**g** Molecule A (green) in the closed form. **h**–**l** Molecule C (salmon) in the open form. In the closed form, Arg267 (the NT region of helix V) hydrogen bonds with Tyr209 (helix III) and Tyr317 (helix VI) as viewed from the intracellular side (**a**), thereby constricting the major cavity (**b**). Pro261 (ICL2) and Arg264 (NT) interact with Lys105 and/or Asp106 of NGD (**c**), Arg264 and Gln265 (NT) interact with Tyr317, Ala318, and Arg320 (helix VI) (**d**), and Gln254 (helix IV) and Asp256 (ICL2) interact with Lys206 (helix III) (**e**) in the closed form. In contrast, in the open form, these interactions are completely disrupted (**j**–**l**). In both forms, Arg257 and Thr260 interact with His263 within ICL2 (**f**, **h**) and Phe258 (ICL2) interacts with Val252 (helix IV) and Thr266 (helix V) (**g**, **i**).

In the closed form, the NT region (Arg264–Arg267) of helix V (Fig. 2b) interacts with helices III and VI and the N-terminal globular domain (NGD). First, the side-chain guanidino group of Arg267, at the end of the NT region, hydrogen bonds with the side-chain hydroxyl groups of Tyr209 (helix III) and Tyr317 (helix VI) (Fig. 4a). Second, the side-chain amino group of Arg264, at the beginning of the NT region, hydrogen bonds with the main-chain carbonyl group of Asp106 from NGD (Fig. 4b). The side-chain methylene groups of Arg264 interact with the phenyl group of Tyr317 (helix VI) and the methylene group of Arg320 (helix VI) (Fig. 4c). Moreover, the side-chain methylene group of Gln265 (helix V) interacts with the methyl group of Ala318 (helix VI) (Fig. 4c).

As the NT region of helix V is firmly fixed, ICL2 (255-WDRFATPKH-263) (Fig. 2b) is close enough to interact with helices III and IV. Thus, the side-chain carboxyl group of Asp256 from ICL2 and the main and side chains of Gln254 at the C-terminal end of helix IV interact with the side-chain amino and methylene groups of Lys206 from helix III (Fig. 4d). The side-chain methylene groups of Pro261 in ICL2 contact the main chains of Lys105 and Asp106 from NGD (Fig. 4b).

Consequently, the NT region of helix V and ICL2 is firmly tied by their strong interactions with the TM helices III, IV, and VI, and NGD. As a result, the M2 region of helix V is kept in the bent conformation even without strong interactions with other TM helices. This indirect effect of the NT region and ICL2 is mediated by the M1 region. In fact, when the helix-V structures of molecules A and B are superimposed at their CT regions, the degrees of the M2-region bending are slightly different from each other, and accordingly the M1 region appreciably deviates between them (Fig. 2e).

Thus, in the closed-form structure, the firmly tied NT region completely closes the inner-leaflet side of the LB opening (Fig. 1d, e, g, h). Concomitantly, the M1 and M2 regions minimize the outer-leaflet side of the LB opening (Fig. 1d, e, g, h). Furthermore, the rigidly positioned ICL2 covers the CP opening almost completely (Fig. 1g, h). Thus, only small LB and CP openings remain on the major cavity.

In contrast, in the open-form structure, the interactions that tie the NT region of helix V and ICL2 to the main body of the protein in the closed form are completely disrupted in the open form (Fig. 4e–g). Consequently, in the open form, the CP and LB openings are drastically enlarged (Fig. 1f, i). Thus, ICL2 protrudes from the CP surface and lacks interactions with other parts of the protein (Supplementary Fig. 3a), and is therefore more mobile than in the closed form.

The other residues of ICL2 are folded similarly between the closed and open forms (Fig. 4h–k), by the intra-loop interactions of the main-chain carbonyl group of Arg257 and the side-chain hydroxyl group of Thr260 with the side-chain imidazole ring of His263 (Fig. 4h, j), and the hydrophobic interactions of Phe258 (ICL2) with Val252 (helix IV) and Thr266 (helix V) (Fig. 4i, k). The folded ICL2 is more extended, concomitant with the bending of helix V, in the closed form than in the open form.

### The closed- and open-form structures of AdipoR1(D208)

**Fig. 5:**
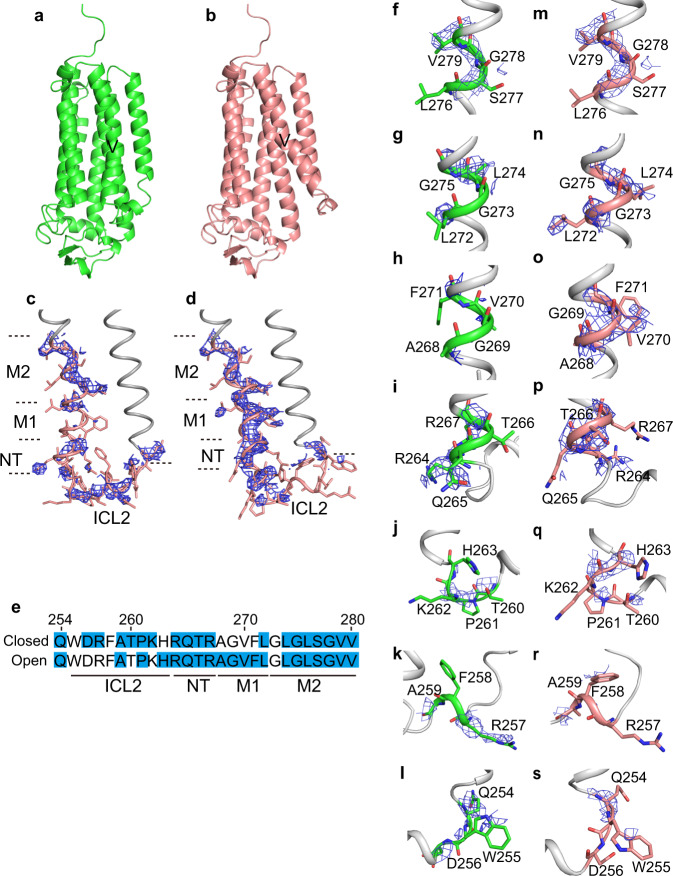
The closed- and open-form structures of AdipoR1(D208). **a**, **b** The closed form (44%) (**a**) and the open form (56%) (**b**) of AdipoR1(D208). **c**, **d** The SA *F*_o_–*F*_c_ omit maps contoured at 2.0*σ* for the closed (**c**) and open (**d**) forms, on the wire models of the dual closed-open conformational regions (residues 250–279), in helix IV, ICL2, and helix V of AdipoR1(A208). The dual-conformational regions are colored salmon, while the remaining parts of the molecules are colored gray. **e** Summary of the residues with electron densities that were observed, fully or partly, in the SA *F*_o_–*F*_c_ omit maps contoured at 2.0*σ*, highlighted in blue in the sequences of the dual-conformational regions. **f**–**s** The SA *F*_o_–*F*_c_ omit maps on helix IV, ICL2, and helix V of molecules A (**f**–**l**) and C (**m**–**s**), contoured at 2.0*σ*. The SA *F*_o_–*F*c omit maps on residues Gln254, Phe255, and Asp256 (**f**, **m**), Arg257, Phe258, and Ala259 (**g**, **n**), Arg264, Gln265, Thr266, and Arg267 (**h**, **o**), Ala268, Gly269, Val270, and Phe271 (**i**, **p**), Thr260, Pro261, Lys262, and His263 (**j**, **q**), Leu272, Gly273, Leu274, and Gly275 (**k**, **r**), and Leu276, Ser277, Gly278, and Val279 (**l**, **s**).

We previously analyzed our diffraction data set for AdipoR1(D208), with one molecule of AdipoR1 in the asymmetric unit^12^. For this single molecule, we could refine the closed-form structure, as the authentic closed-form structure of AdipoR2(D219) had been determined^12^. On the other hand, the same data set was recently reanalyzed by another group, assuming the open form^13^ (PDB ID 5LXG) with no authentic open-form structure. As we now have the reliable structures of both the authentic closed and open forms for AdipoR1(A208), we reanalyzed the previous AdipoR1(D208) diffraction data set, and refined the structure as a mixture of the closed and open forms, assuming the dual conformations for residues 250–279. Thus, in the previous crystal, 44% and 56% of the AdipoR1(D208) molecule assume the closed and open forms, respectively (Fig. 5, Table 1, PDB ID 6KRZ).

The closed- and open-form structures of AdipoR1(D208) refined on the assumption of the dual closed-open conformation (Fig. 5a, b) are quite similar to those of molecules A/B and C, respectively, of AdipoR1(A208). The r.m.s.d. values for the main-chain Cα atoms are 1.006 and 0.705 Å for the closed form of AdipoR1(D208) against molecules A and B, respectively, of AdipoR1(A208), and 0.806 Å for the open form of AdipoR1(D208) against molecule C of AdipoR1(A208).

We examined this result for the dual-conformational region (residues 250–279) in helices IV and V and ICL2, by using the simulated-annealing (SA) *F*_o_–*F*_c_ omit maps at 2.5*σ* and 2.0*σ* (Fig. 5c, d, f–s, Supplementary Fig. 7a–d), prepared with phenix.refine^19^. The electron densities corresponding to both of the closed- and open-form polypeptide chains, positioned distinctly between the two forms, were certainly observed for the dual-conformational region of AdipoR1(D208) (Fig. 5c, d). These observations are in sharp contrast to the SA *F*_o_–*F*_c_ omit maps for molecules A–C of AdipoR1(A208), in which the electron densities were predominantly observed on the closed form for molecules A and B and on the open form for molecule C (Fig. 1j–l, Supplementary Fig. 8a, c, e, g, j, l). In contrast, only the noise-level electron densities were observed on the open form for molecules A and B and on the closed form for molecule C in the SA *F*_o_–*F*_c_ omit maps (Supplementary Fig. 8b, d, f, h, i, k). Naturally, in the SA *F*_o_–*F*_c_ omit maps of AdipoR1(D208), the electron densities for the dual-conformational region are weaker than those for the rest of the molecule, due to their 445 and 56% occupancies, respectively. Nevertheless, the electron densities on the closed and open forms are both far above the noise level (Fig. 5c, d, Supplementary Fig. 7a–d). We will describe the SA *F*_o_–*F*_c_ omit map at 2.0*σ*.

For comparison, we examined the omit maps for the structure of AdipoR (D208) refined on the assumption of the open form only^13^ (PDB ID 5LXG), prepared with phenix.refine and autoBuster. The electron densities were surely observed on the closed form (Supplementary Figs. 7e–h and 9a) as well as on the open form, similarly to our case (Fig. 5c, d, Supplementary Fig. 7a–d). In the single-conformation open-form structure (PDB ID 5LXG), nine water molecules and the Phe271 side chain are modeled within the electron densities corresponding to the closed form (Supplementary Fig. 9b, c), but hardly account for the entire closed-form electron density (Supplementary Fig. 9d–g).

### Distinct conformational characteristics of ICL2 and the NT, M1, and M2 regions of helix V between the closed and open forms of AdipoR1(D208)

In the SA *F*_o_–*F*_c_ omit maps for AdipoR1(D208), the electron densities corresponding to Leu274–Val280 in the M2 region were continuously observed along both the inward-bent and straight 3_10_-helical structures in the closed and open forms, respectively (Fig. 5c, d, f, g, m, n), which unambiguously confirms the dual closed-open conformation. On the other hand, the electron densities corresponding to the other regions of helix V and ICL2 were somewhat discontinuous or fragmented in distinct manners between the closed and open forms (Fig. 5c, d, h–l, o–s): the positions of the clearly and poorly observed electron densities are quite different between the two forms, as summarized in Fig. 5e. These electron density patterns for the closed and open forms of AdipoR1(D208) correspond well to the characteristics of the conformations and interactions observed for molecules A/B and C, respectively, of AdipoR1(A208), as follows.

In the closed form of molecule A, ICL2 (Trp255–His263) and the NT region (Arg264–Gln267) of helix V are firmly fixed to NGD and helices III and VI (Fig. 4a–d). Correspondingly, in the SA *F*_o_–*F*_c_ omit map of AdipoR1(D208) for the closed form (Fig. 5c, f–l), the electron densities of ICL2 and the NT region of helix V are clear, particularly for the residues interacting with the main body of the protein. First, the electron density of Arg267 in the NT region is obvious (Fig. 5i), as the side chain of Arg267 interacts with Tyr209 of helix III and Tyr317 of helix VI (Fig. 4a). Second, the main- and side-chain electron densities of Arg264 and Gln265 at the beginning of helix V are unambiguous (Fig. 5i), as the side chain of Arg264 interacts intensively with Asp106 of NGD, and with Tyr317 and Arg320 of helix VI, and the side chain of Gln265 interacts with Ala318 of helix VI (Fig. 4b, c). Third, the main- and side-chain electron densities of Gln254 at the end of helix IV and Asp256 in ICL2 are clearly observed (Fig. 5l), while the main and side chains of Gln254 and the side chain of Asp256 interact with the side chain of Lys206 from helix III (Fig. 4d). Furthermore, the electron density of Arg257 is clearly observed (Fig. 5k), as this residue is involved in the intra-loop interaction (Fig. 4h).

In contrast, in the open form, neither ICL2 nor the NT region of helix V has such binding interactions (Fig. 4j–l). Correspondingly, in the SA *F*_o_–*F*_c_ omit map of AdipoR1(D208) for the open form (Fig. 5d, m–s), the electron densities for ICL2 are much less intensively observed, as compared with those in the closed form. In more detail, the electron densities of residues 255–258 are barely visible (Fig. 5e, r, s), and only the main-chain electron densities are observed for Gln254 at the end of helix IV (Fig. 5s) and Arg264 and Gln265 at the beginning of helix V (Fig. 5p) in the open form.

On the other hand, in the SA *F*_o_–*F*_c_ omit map of AdipoR1(D208) for the open form, the electron densities for the M1 region (Ala268–Gly269–Val270–Phe271–Leu272) of helix V are clear (Fig. 5n, o). This observation corresponds very well to the extensive hydrophobic interactions of Val270 and Phe271 of the M1 region with helices III and/or IV in the open form of AdipoR1(A208) (Fig. 3d–f), which predominantly define the degree of bending and orientation of helix V in the open form (Fig. 2a, d, e, j). Concomitantly, the electron densities for the NT region of helix V are easily visualized, probably because the NT and M1 regions form a straight coaxial α-helix. In contrast, in the SA *F*_o_–*F*_c_ omit map of AdipoR1(D208) for the closed form, the electron densities for one turn (Ala268–Gly269–Val270–Phe271) of the M1 region of helix V are barely distinguishable (Fig. 5c, h). This phenomenon may be explained by the characteristic conformational properties of helix V in the closed form. First of all, the specific inward-bent structure of helix V (Fig. 2a, c–e, i) is determined by the extensive interactions of ICL2 and the NT region of helix V with NGD and helices III and VI (Fig. 4a–d) in the closed form. Actually, the position of the M1 region deviates appreciably between molecules A and B in the closed form (Fig. 2e), probably because of the high plasticity of the M2 region of helix V (Fig. 2i).

In summary, for the residues in the former half of ICL2 and the NT and M1 regions of helix V, the electron densities could be observed for their parts (main and/or side chains) that are fixed rigidly to the main body of the protein by interactions with helices III, IV, and/or VI, and/or NGD (Figs. 3–5). For other residues in the two regions; that is, the latter half of ICL2 (Ala259–His263, Fig. 5e, j, k, q, r) and throughout the M2 region, except for Gly273 (Leu274–Val280, Fig. 5e, f, g, m, n), the electron densities are clearly visible in the SA *F*_o_–*F*_c_ omit map of AdipoR1(D208), in quite similar manners with respect to their local conformations between the closed and open forms, although their spatial locations relative to the main body of the protein are distinct (Figs. 2a–e, i, j and 4h–k, Supplementary Figs. 5 and 6).

Thus, the SA *F*_o_–*F*_c_ omit map exhibits the electron densities corresponding to both the closed and open forms, accurately reflecting the distinct characteristic interactions of ICL2 and the NT region of helix V in the closed form, and the M1 region in the open form, with other parts of the protein to determine the bending and orientation of helix V. Overall, these results unambiguously demonstrate that AdipoR1(D208) in the crystal adopts the dual conformations corresponding to the closed and open forms of AdipoR1(A208).

## Discussion

In this study, we determined the crystal structure of AdipoR1(A208), which is fully active with respect to the downstream AMP kinase activation, and observed the closed-form structure for two of the three independent molecules and the open-form structure for the other one in the asymmetric unit. On the basis of these two authentic structures of the closed and open forms, we reanalyzed our previous diffraction data set of AdipoR1(D208) and determined that it represents a dual conformation consisting of the closed form (44%) and the open form (56%). These observations indicate that AdipoR1 can assume both the closed and open forms.

In both the closed and open forms of AdipoR1, the zinc ion exhibits tetrahedral coordination with the three conserved His residues, His191, His337, and His341 (Supplementary Fig. 2a–c). There are two conserved residues, Ser187 and Asp208, in the proximity, and they may interact through water molecules with the zinc ion^12^. Interestingly, none of the single mutations, His191Ala, Asp208Ala, His337Ala, and His341Ala, affected the AdipoR1 activity to activate the downstream AMP kinase, although the quadruple mutant His191Ala/Asp208Ala/His337Ala/His341Ala decreased the activity^12^. Therefore, the zinc coordination, or the putative hydrolytic activity^12,13^, of AdipoR1 is not essentially required for AMPK activation, which is the major downstream signaling pathway of AdipoR1. However, the zinc ion binds helices II, III, and VII together, and probably stabilizes the structure of the subdomain consisting of helices I, II, III, and VII^12^. Given the structural stabilization by the zinc ion, AdipoR1 may retain its native overall structure, even while helices IV and V dynamically exchange between the closed and open forms.

The closed and open forms of AdipoR1 may be related to the adiponectin receptor functions, as follows. First, between the open- and closed-form structures, the positions of the CP ends of helices IV and V are substantially different, by 3.6 and 11 Å, respectively. Thereby, the position of ICL2 (Trp255–His263), which connects helices IV and V, differs by as much as 13.5 Å between the closed and open forms (Supplementary Fig. 3a). This change in the ICL2 position, coupled with the closed-open transition of AdipoR1, might be relevant to the downstream signaling. First, the entire ICL2 is protruded and much more exposed in the open form than in the closed form. Furthermore, the ICL2 loop of AdipoR1 has three positively charged residues, Arg257, Lys262, and His263, whereas that of AdipoR2 has uncharged residues, Met, Gln, and Tyr, respectively, at the corresponding positions (Supplementary Fig. 3b, c). These positively charged side chains are oriented outward in the open form, but less so in the closed form. In this context, the downstream signaling pathways are quite distinct between the two adiponectin receptors: AdipoR1 activates the AMPK pathways^2–4^, and AdipoR2 activates the PPAR-α pathways, with increased expression of UCP2^5–7^. AdipoR1, but not AdipoR2, reportedly interacts with the scaffolding proteins RACK1 and Cav-3 and the cyclin-dependent kinase CK2*β*^20–22^. Therefore, the hypothesis that the ICL2 conformation change is relevant to the downstream signaling may explain the distinct downstream signaling pathways between AdipoR1 and AdipoR2.

## Methods

### Preparation of AdipoR1(A208) crystals

The AdipoR1(A208) protein and the Fv fragment of an anti-AdipoR1 monoclonal antibody were prepared, as described^11,12^. In brief, human AdipoR1 (residues 89–375) with the D208A mutation was expressed in HEK293 cells^23^. The AdipoR1(A208) protein was purified by FLAG antibody affinity chromatography, followed by anion exchange chromatography. The Fv fragment was synthesized as a fusion protein with His and SUMO tags by the *Escherichia coli* cell-free protein synthesis method, and was purified by Ni-affinity chromatography, followed by Ni-affinity chromatography after SUMO tag cleavage with His-tagged SUMO protease, and size-exclusion chromatography. The purified AdipoR1(A208) protein was mixed with the Fv fragment, and the AdipoR1(A208)–Fv complex was purified by size-exclusion chromatography. The purified Fv-bound AdipoR1(A208) was reconstituted into the monoolein lipidic mesophase, at a ratio of 40:6:54 (w/w) for protein:cholesterol:monoolein. The resulting lipidic mesophase sample was dispensed into 96-well glass plates in 30–40 nl drops, overlaid with 800 nl precipitant solution, and covered with thin cover glasses, by the use of laboratory-constructed robotic microdispensers^15^. Crystallization setups were performed at room temperature, and the plates were incubated at 20 °C. The crystals of AdipoR1(A208) were grown in precipitant conditions of 30–32% (w/v) PEG 400 in 100 mM Tris-HCl buffer (pH 7.5), containing 100–150 mM lithium acetate. Crystals were harvested directly from the lipidic mesophase using MiTeGen micromounts, and flash cooled in liquid nitrogen.

### X-ray data collection

Data collection for AdipoR1(A208) was performed on beamline BL32XU at SPring-8 by the helical scan method, with a beam size of 1 μm × 12 μm (horizontal × vertical) using 1° oscillation^24,25^. Diffraction data of AdipoR1(A208) were collected from a single crystal. The data from the AdipoR1(A208) crystal were indexed, scaled, and merged with the XDS package^26^ and AIMLESS^27^. Diffraction images were collected using an MX225HS CCD detector (Rayonix, LLC). The data collection statistics are summarized in Table 1. The AdipoR1(A208) crystals belonged to the space group *P*2_1_2_1_2_1_, with unit-cell parameters *a* = 104.59, *b* = 119.35, and *c* = 197.81 Å.

### Structure solution and refinement

The crystal structure of AdipoR1(A208) was determined by molecular replacement with Phaser^28^, using the PDB structure 3WXV as the search model. Refinement was performed with phenix.refine^19^, and the refined coordinates were rebuilt manually with COOT^29^. The crystal structure of AdipoR1(A208) was refined with final *R*_work_/*R*_free_ values of 0.2192/0.2722. The final models of the three molecules of AdipoR1(A208) in the asymmetric unit include 284, 282, and 282 residues (molecules A, B, and C, respectively) of the receptor, 357 residues of V_H_, and 321 residues of V_L_. For 6KRZ, 96.42% of the residues are in the Ramachandran favored regions. The data collection and refinement statistics are summarized in Table 1. Structural illustrations were generated using PyMOL^30^.

The previous data set of AdipoR1(D208) (PDB ID 3WXV) was reanalyzed on the assumption of the dual conformations for helices IV and V and ICL2 in the closed and open forms. The domain occupancy optimization of helix IV–ICL2–helix V (residues 250–279) was performed with phenix.refine, and the ratio of the closed and open conformations was 44:56. We started the occupancy refinement from 70:30, 50:50, and 30:70 separately, and in all cases reached the final score of 44:56. The same ratio was observed when we tried the structure determination by molecular replacement with the single, open-form AdipoR1 structure (PDB ID 5LXG) as the search model. By intensive examination of the *F*_o_–*F*_c_ SA omit maps, the assumption of the dual conformations was confirmed to be correct.

For the present dual closed-open structure, the final *R*_work_/*R*_free_ values with phenix.refine are 0.2188/0.2755, while the *R*^xpct^_work_/*R*^xpct^_free_ values with autoBUSTER^31^ are 0.199/0.268. On the other hand, for the previous single open-form structure (PDB ID 5LXG), the final *R*_work_/*R*_free_ values with phenix.refine are 0.2208/0.2606, while the *R*^xpct^_work_/*R*^xpct^_free_ values with autoBuster are 0.208/0.254. The latter values, 0.208/0.254, were obtained according to the standard parameters (five cycles of TLS refinement). In contrast, different values of 0.227/0.235 were obtained with the nonstandard parameters (single cycle of refinement), which are close to the values of 0.225/0.234 reported in the PDB. Therefore, the simple comparison of the final *R*_work_/*R*_free_ values between the single and dual-conformational assumptions does not clearly show which is superior. As a consequence, we decided to rely on the above-mentioned examination of the *F*_o_–*F*_c_ SA omit maps, which supported the dual-conformational assumption. The final structures of the dual closed-open structures have been deposited in the PDB, with the accession code 6KS0, by replacing 3WXV. For 6KS0, 95.85% of the residues are in the Ramachandran favored regions.

The major differences between the previous single open-form structure (5LXG) and the present dual closed-open structure are as follows. In the former structure, the open form is modeled with the full occupancy and the nine water molecules are modeled in the positions corresponding to the closed form. In the latter structure, the open form was modeled without the nine water molecules with 66% occupancy, and the closed form was modeled with 44% occupancy. In addition, at least 20 water molecules were modeled on the outer surface of the TM region of AdipoR1 in 5LXG. Since these positions correspond to the hydrophobic part of the LB, in the present study we did not model any water molecules in this location. Therefore, for direct comparison with our dual closed-open structure, we reevaluated the *R*_work_/*R*_free_ and *R*^xpct^_work_/*R*^xpct^_free_ values of the single open-form structure (5LXG), by using the same set of water molecules as that in ours, but retaining the nine water molecules that they modeled in the positions corresponding to the closed form. The obtained *R*_work_/*R*_free_ and *R*^xpct^_work_/*R*^xpct^_free_ values are 0.2357/0.2799 and 0.223/0.267, respectively. On the other hand, the corresponding values for our dual closed-open structure are 0.2209/0.2773 and 0.198/0.269, respectively, as described above. Thus, our dual closed-open structure exhibits nearly the same *R*_free_/*R*_free_ values and obviously smaller *R*^xpct^_work_/*R*^xpct^_work_ values, as compared with the single open-form structure.

The previous data set of AdipoR2(D219) (PDB ID 3WXW) was also reanalyzed, including one oleic acid molecule. The final closed-form structure has been deposited in the PDB, with the accession code 6KS1, by replacing 3WXW. For 6KS1, 97.43% of the residues are in the Ramachandran favored regions. The *R*_work_/*R*_free_ values for our revised structure (6KS1) are 0.2256/0.2640. On the other hand, the *R*_work_/*R*_free_ values for the previously revised structure (5LWY) are 0.2288/0.2673, including 113 water molecules in 5LWY modeled either within the space corresponding to the LB or with very weak electron density (*σ* < 0.8) in the 2*F*_o_–*F*_c_ map. When the 113 improper water molecules were removed from 5LWY, most of the remaining water molecules were the same as those in our reanalyzed structure (6KS1), and the *R*_work_/*R*_free_ values became 0.2224/0.2642 and further closer to ours (0.2256/0.2640). We also (re)evaluated, with the standard parameters, the *R*^xpct^_work_/*R*^xpct^_free_ values and obtained 0.185/0.239 for our revised structure, and 0.186/0.233 and 0.191/0.231 for the previously revised structure (5LWY) with and without the 113 improper water molecules, respectively.

### Analyses of free fatty acids in the AdipoR1 and AdipoR2 preparations

Free fatty acids in the AdipoR1(A208), AdipoR1(D208), AdipoR2(A219), and AdipoR2(D219) preparations from HEK293 cells, and the AdipoR1(D208) and AdipoR2(D219) preparations from High Five cells were analyzed as follows. The lipids in the samples were dissolved in methanol. The liquid chromatography-tandem mass spectrometry (LC-MS/MS) system consisted of an UltiMate 3000 HPLC (Thermo Fisher Scientific, San Jose, CA), a C18-based reverse-phase column (SHISEIDO), and a TSQ Quantiva triple quadrupole mass spectrometer (Thermo Fisher Scientific, San Jose, CA), equipped with a heated-electrospray ionization-II source. The LC-MS/MS system was used with slight modifications of the methods described previously^20^: the LC separation was performed using gradient elution with solvent A (5 mM ammonium formate in water) and solvent B (5 mM ammonium formate in 95% (v/v) acetonitrile) at 200 μL/min. The initial condition was set at 30% B. The following solvent gradient was applied: maintain 30% B for 1 min, followed by a linear gradient to 80% B from 1 to 5 min, then 100% B from 5 to 10 min, and hold at 100% B for 5 min, then 30% B from 15 to 16 min, and finally maintain 30% B for 4 min. The MS detection mode was selected ion monitoring.

### Statistics and reproducibility

The purification of AdipoR1(A208) was repeated several times, which showed same results. The X-ray data collection and refinement statistics were summarized in Table 1. The statistical analyses used in mass spectrometry are presented in the legend of Supplementary Fig. 10.

### Reporting summary

Further information on research design is available in the Nature Research Reporting Summary linked to this article.

## Supplementary information

Supplementary Information

Reporting Summary

## Data Availability

The coordinates and structure factors have been deposited in the Protein Data Bank: the two closed-form structures and the open-form structure of AdipoR1(A208) (accession code 6KRZ), the closed-form structure (44%) and the open-form structure (56%) of AdipoR1 (accession code 6KS0, replacing 3WXV), and the closed-form structure of AdipoR2 with oleic acid (accession code 6KS1, replacing 3WXW). Other data are available from the corresponding authors upon reasonable request. Readers are welcome to comment on the online version of the paper.
