# Supplementary material for: Human adiponectin receptor AdipoR1 assumes closed and open structures
Source: Commun Biol. 2020 Aug 14;3:446. doi: 10.1038/s42003-020-01160-4 (PMC7427782; doi:10.1038/s42003-020-01160-4)
Supplement: Supplementary file 1 — Supplementary Information [file 42003_2020_1160_MOESM1_ESM.pdf]

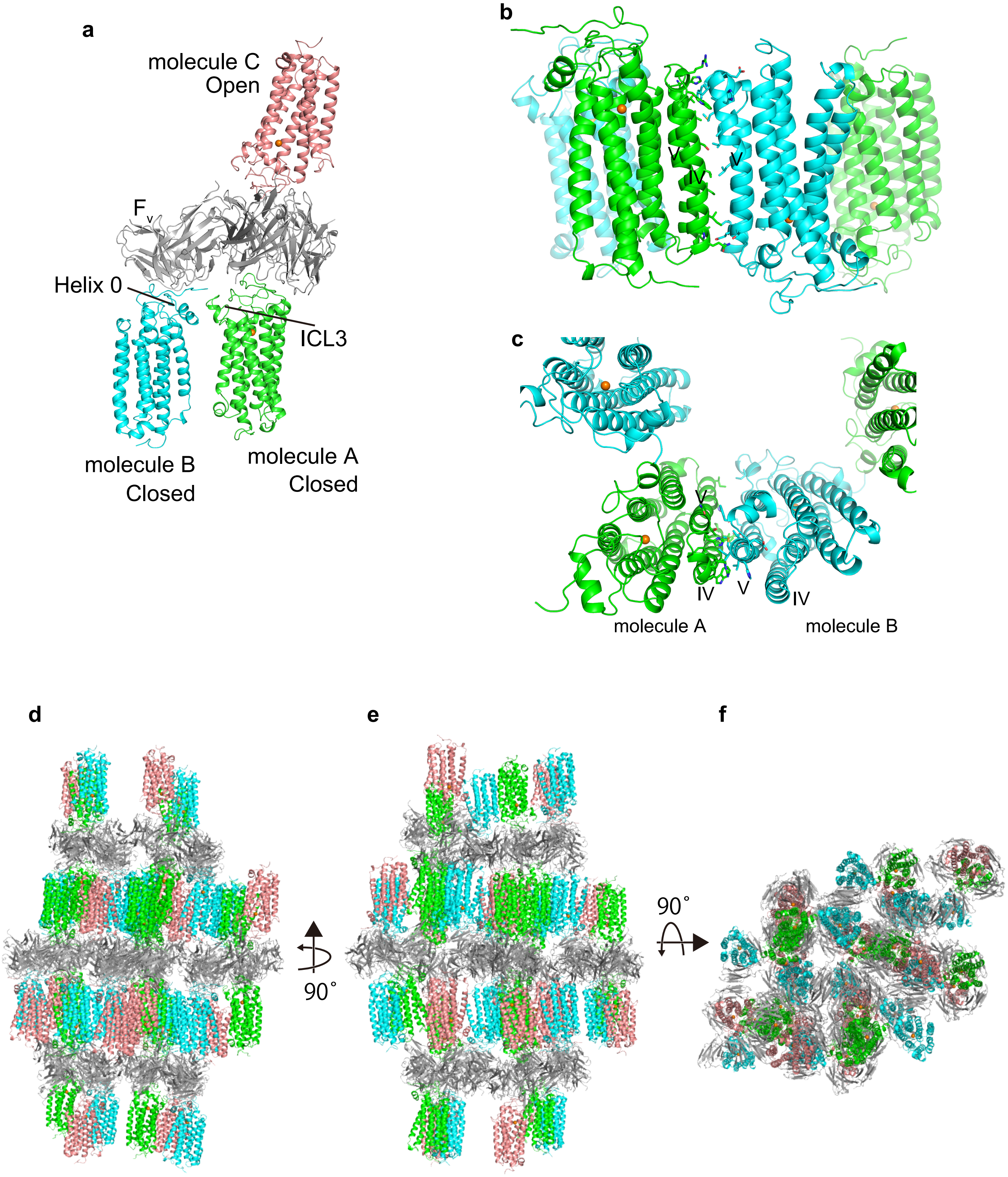
a
molecule C
Open
Fv
Helix 0
ICL3
molecule B
Closed
molecule A
Closed
b
V
IV
V
c
V
IV
V
IV
molecule A
molecule B
d
e
f
90˚
90˚

Supplementary Figure 1 | Crystal packing of AdipoR1 (A208)

a, Molecules A (green), B (cyan), and C (salmon) in the asymmetric unit. The Fv fragments are colored gray. The zinc ion is shown as an orange sphere. Molecules A (green) and B (cyan) are in the closed form, and molecule C (salmon) is in the open form. ICL3 of molecule A and helix 0 of molecule B interact with each other. **b**, **c**, Interactions of helices IV and V and ICL2 of molecule A with helix V of the symmetry-related molecule B, viewed parallel to the membrane (**b**) and on the extracellular side (**c**). The interacting residues are shown as sticks. The zinc ion is shown as an orange sphere. **d**–**f**, Lattice packing of the AdipoR1(A208) crystal. AdipoR1 molecules A, B, and C and Fv are colored green, cyan, salmon, and gray, respectively.

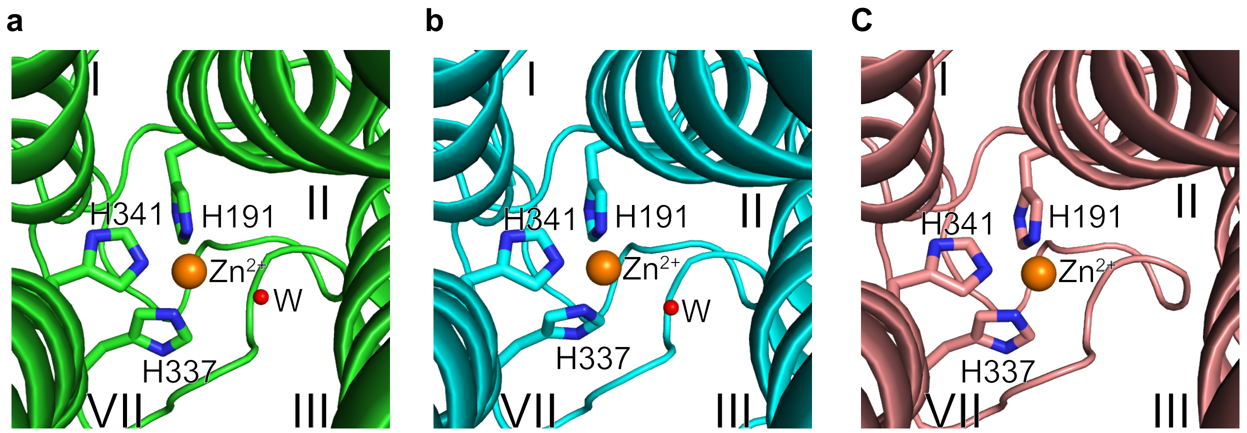

Supplementary Figure 2 | Coordination of the zinc ion in AdipoR1(A208)

The Zn ion and the coordinating His residues in the crystal structure of AdipoR1(A208). Molecule A (**a**, green), molecule B (**b**, cyan), and molecule C (**c**, salmon) are viewed from the extracellular side. The zinc ion and a water molecule are shown as orange and red spheres, respectively. The three conserved His residues, His191, His337, and His341, are shown as stick models.

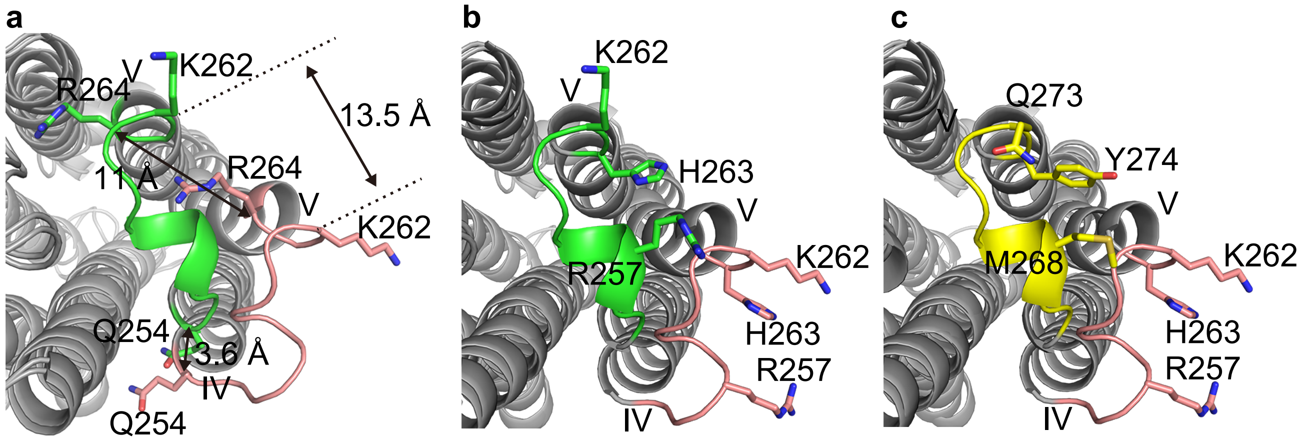

d

R1 255 WDRFATPKH 263
R2 266 WDMFATPQY 274

Supplementary Figure 3 | Comparison of the ICL2 conformations between the closed- and open-form structures of AdipoR1(A208) and/or AdipoR2(D219)

a–**c**, The ICL2 conformations of molecule A (green, closed) and molecule C (salmon, open) of AdpoR1(A208) (**a**, **b**) and AdipoR2(D219) (yellow, closed) and molecule C (salmon, open) of AdpoR1(A208) (**c**), viewed from the intracellular side. Stick models are shown for Gln254, Lys262, and Arg264 of AdipoR1 in **a**, Arg257, Lys262, and His263 of AdipoR1 in **b** and **c**, and Met268, Gln273, and Tyr274 of AdipoR2 in **c**. **d**, The amino acid sequences of the ICL2 regions of AdipoR1 and AdipoR2. The different amino acids between AdipoR1 and AdipoR2 are colored red.

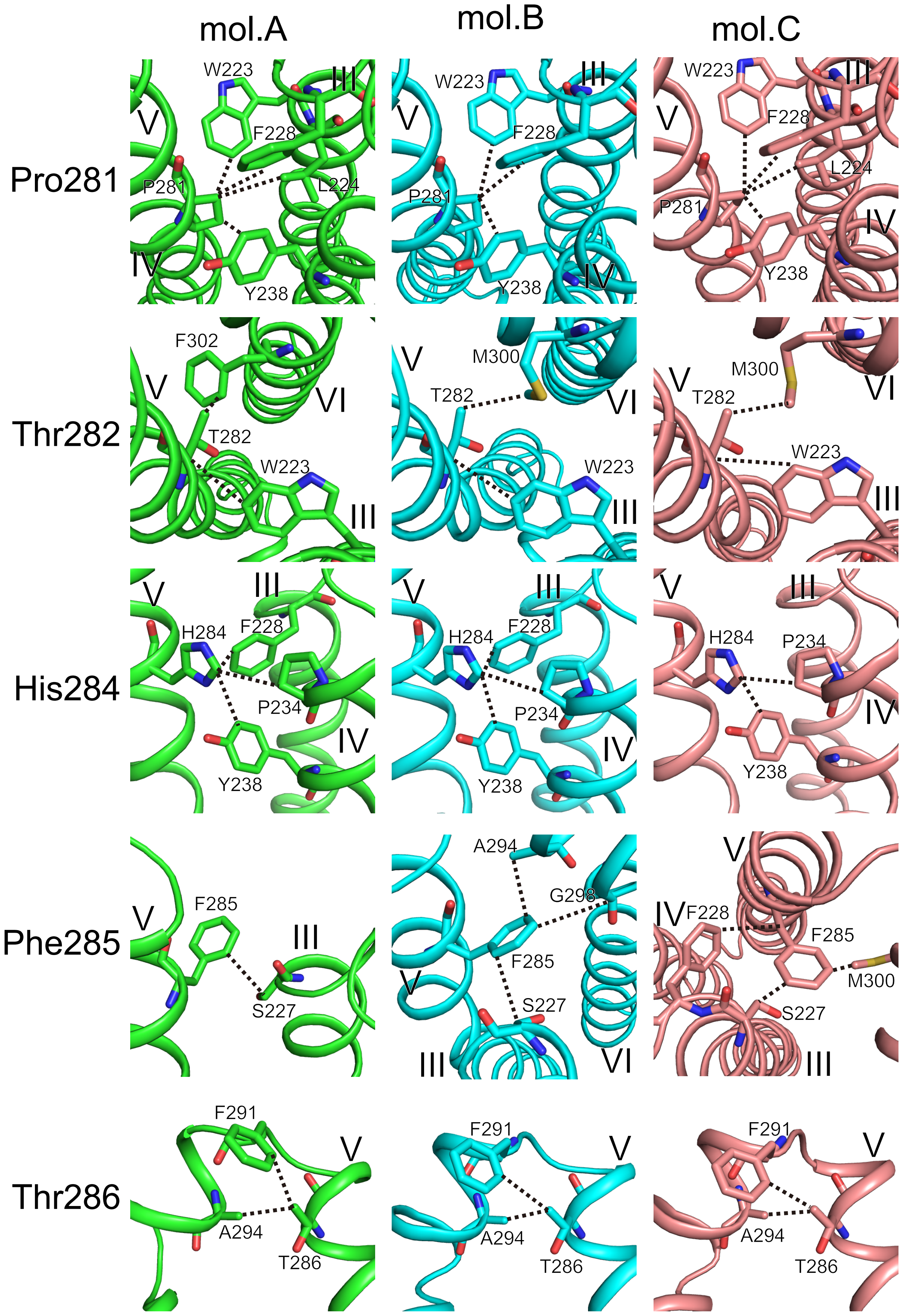

Tanabe et al. Supplementary Figure 4

Supplementary Figure 4 | Interactions of the C-terminal part of helix V with other helices in molecules A–C of AdipoR1(A208)

Pro281, Thr282, His284, Phe285, and Thr286 (stick models) in the C-terminal part of helix V (cartoon) with the interacting residues (stick models) from helices III, IV, and/or VI (cartoons) are shown for molecule A (mol. A, green), molecule B (mol. B, cyan), and molecule C (mol. C, salmon). Direct interactions are indicated with dashed lines. Helices not interacting with the C-terminal part of helix V are omitted for clarity.

Tanabe et al. Supplementary Figure 4

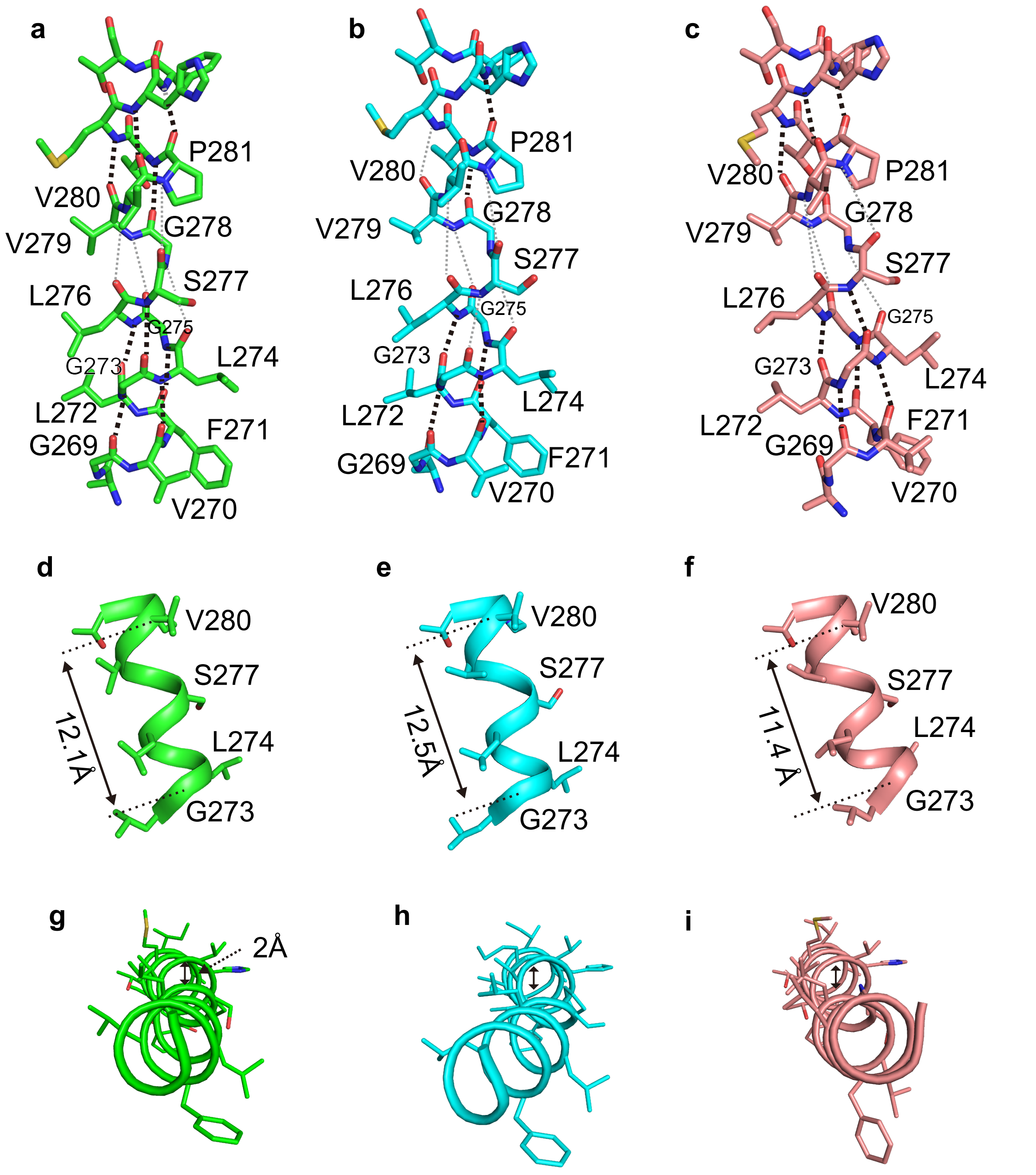

Supplementary Figure 5 | Conformations of helix V in molecules A–C of AdipoR1(A208)
a–**c**, The main-chain C=O : H–N hydrogen bonding for the residues from Gly269 to Pro281 of helix V in molecule A (green, closed) (**a**), molecule B (cyan, closed) (**b**), and molecule C (salmon, open) (**c**). The main-chain oxygen atom of residue *i* and the main-chain nitrogen atom of residue *i*+4 are connected with a thick dashed line when the O–N distances are shorter than or equal to 3.5 Å, and with a thin dashed line for the others. **d**–**f**, Narrow winding of the turns from Gly273 to Val280 in the M2 region of helix V of molecules A (**d**), B (**e**), and C (**f**), which is relevant to the 3_{10}-helical conformation supported by weak main-chain C=O : H–N hydrogen bonds, and results in the longer advance for Gly273–Val280 (11.4–12.5Å) than that of the standard helix (10.5 Å). **g**–**i**, Dislocation of the axes of the α-helices in the M1 and CT regions by 2 Å due to the pliable M2 region, which is bent in the closed form (molecules A (**g**) and B (**h**) and straight in the open form (molecule C) (**i**).

Tanabe et al. Supplementary Figure 5

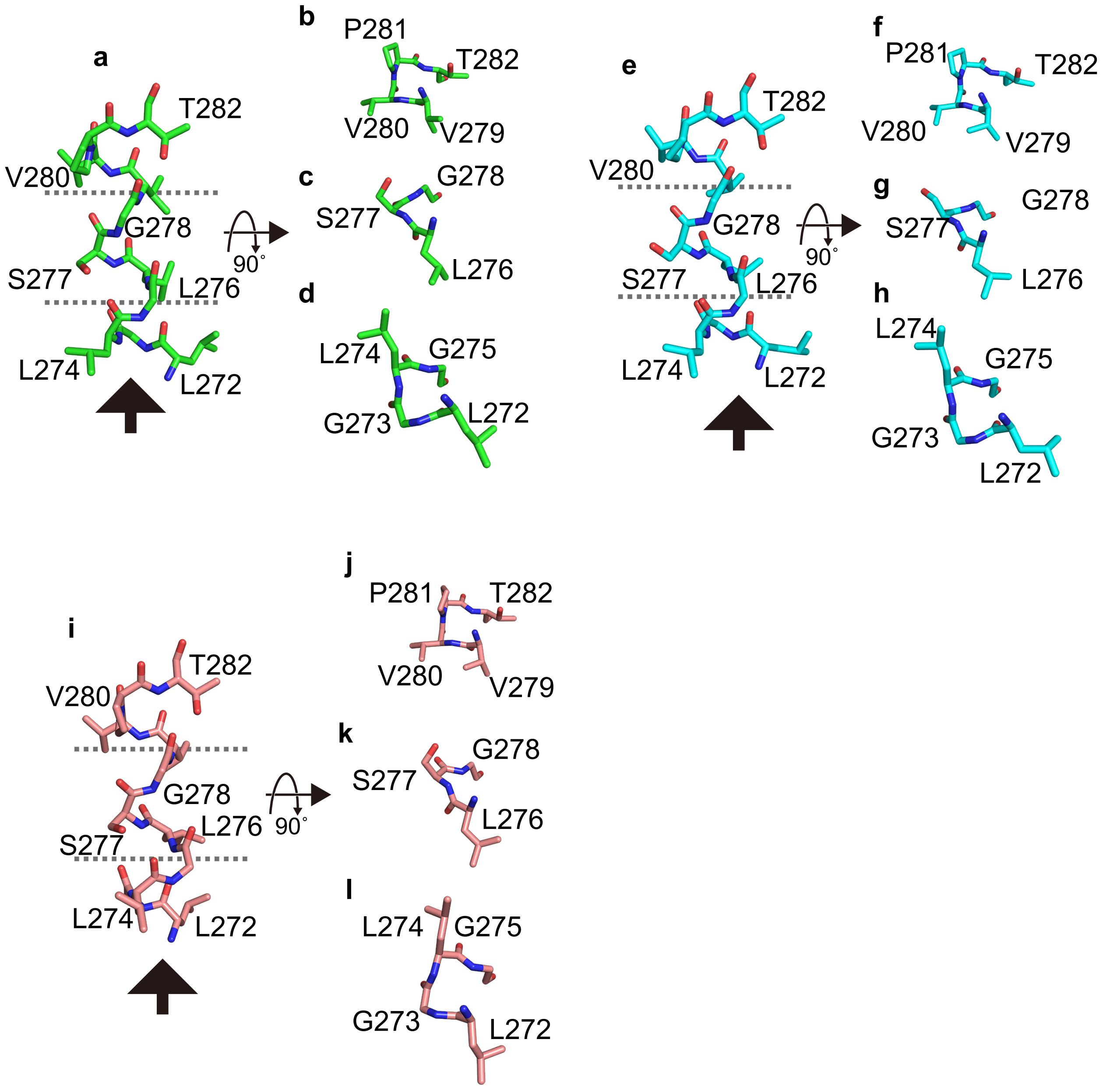

Supplementary Figure 6 | Conformation of Leu272–T282 in helix V in the closed and open forms of AdipoR1(A208)

Stick models of Leu272–T282 in molecules A (**a**–**d**), B (**e**–**h**), and C (**i**–**l**) of AdipoR1(A208), viewed parallel to (**a**, **e**, **h**) and along (**b**–**d**, **f**–**h**, **j**–**l**) the membrane. The 3_{10}-helical conformation occurs in the M2 region (Gly273–Val280) of helix V, in which the central residues, Leu276, Ser277, and Gly278, assume the typical winding of a 3_{10}-helix (three residues per turn) (**c**, **g**, **k**).

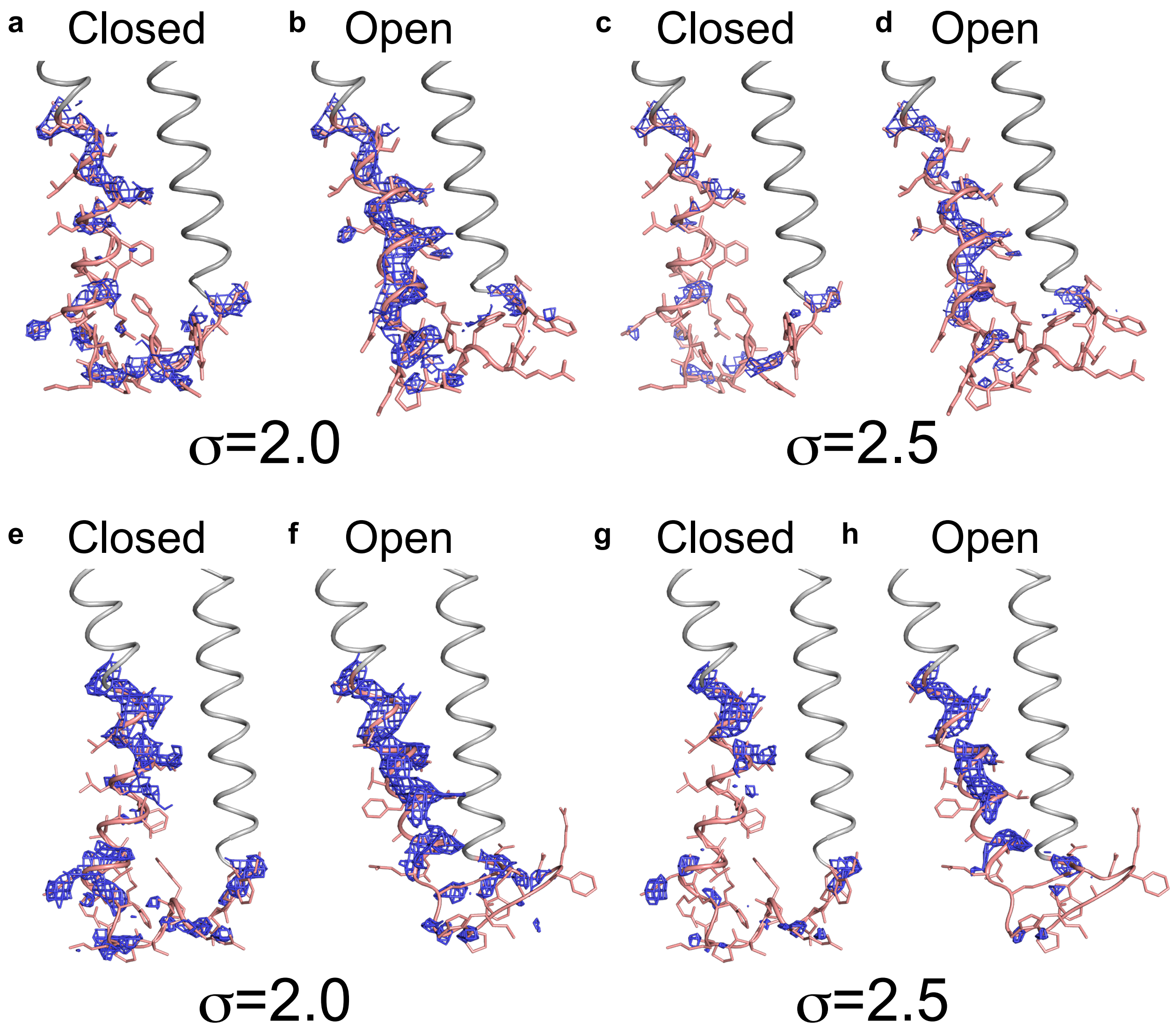

Supplementary Figure 7 | The simulated-annealing *F*o–*F*c omit maps on helices IV and V and the ICL2s of AdipoR1(D208)

The simulated-annealing *F*o–*F*c omit maps of AdipoR1(D208) on the assumptions of the dual conformations (**a**–**d**) and single (**e**–**h**) conformation (PDB IDs 6KS0 and 5LXG, respectively), contoured at 2.0 (**a**, **b**, **e**, **f**) and 2.5 σ(**c**, **d**, **g**, **h**), on residues 254–279 from the middle of helix V, through ICL2, to the middle of helix IV (salmon cartoon). The other parts of helices IV and V (residues 230–253 and 280–290, respectively) are also shown as gray cartoons. Regardless of the assumption (dual or single), two traces of the electron density were observed corresponding to residues 254–279, one in the closed form (**a**, **c**) and the other in the open form (**b**, **d**), with rough occupancies of 44% and 56%, respectively (PDB ID 6KS0).

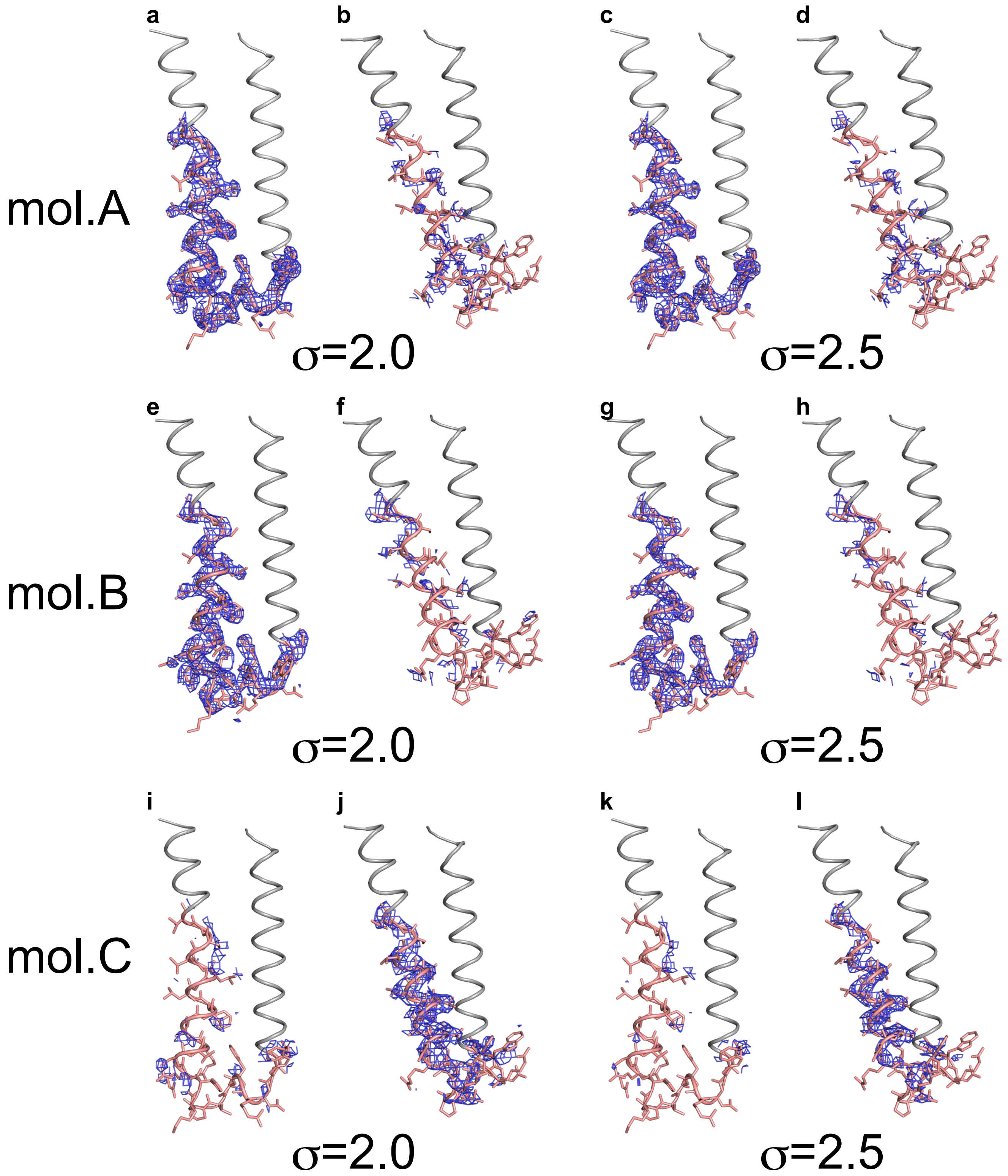

Supplementary Figure 8 | The simulated-annealing *F*o–*F*c omit maps on helices IV and V and the ICL2s of AdipoR1(A208)

The simulated-annealing *F*o–*F*c omit maps, contoured at 2.0 (**a**, **b**, **e**, **f**, **i**, **j**) and 2.5 σ (**c**, **d**, **g**, **h**, **k**, **l**), on residues 251–280 in the closed form (**a**, **c**, **e**, **g**, **i**, **k**) and the open form (**b**, **d**, **f**, **h**, **j**, **l**) for molecules A (**a**–**d**), B (**e**–**h**), and C (**i**–**l**) of AdipoR1 (A208) (PDB ID 6KRZ). Residues 254–279 spanning from the middle of helix V, through ICL2, to the middle of helix IV are shown as salmon cartoons, together with the remaining parts of helices IV and V (residues 230–253 and 280–290, respectively) as gray cartoons. The electron densities are observed predominantly on the closed form for molecules A and B (**a**, **c**, **e**, **g**) and on the open form for molecule C (**j**, **l**).

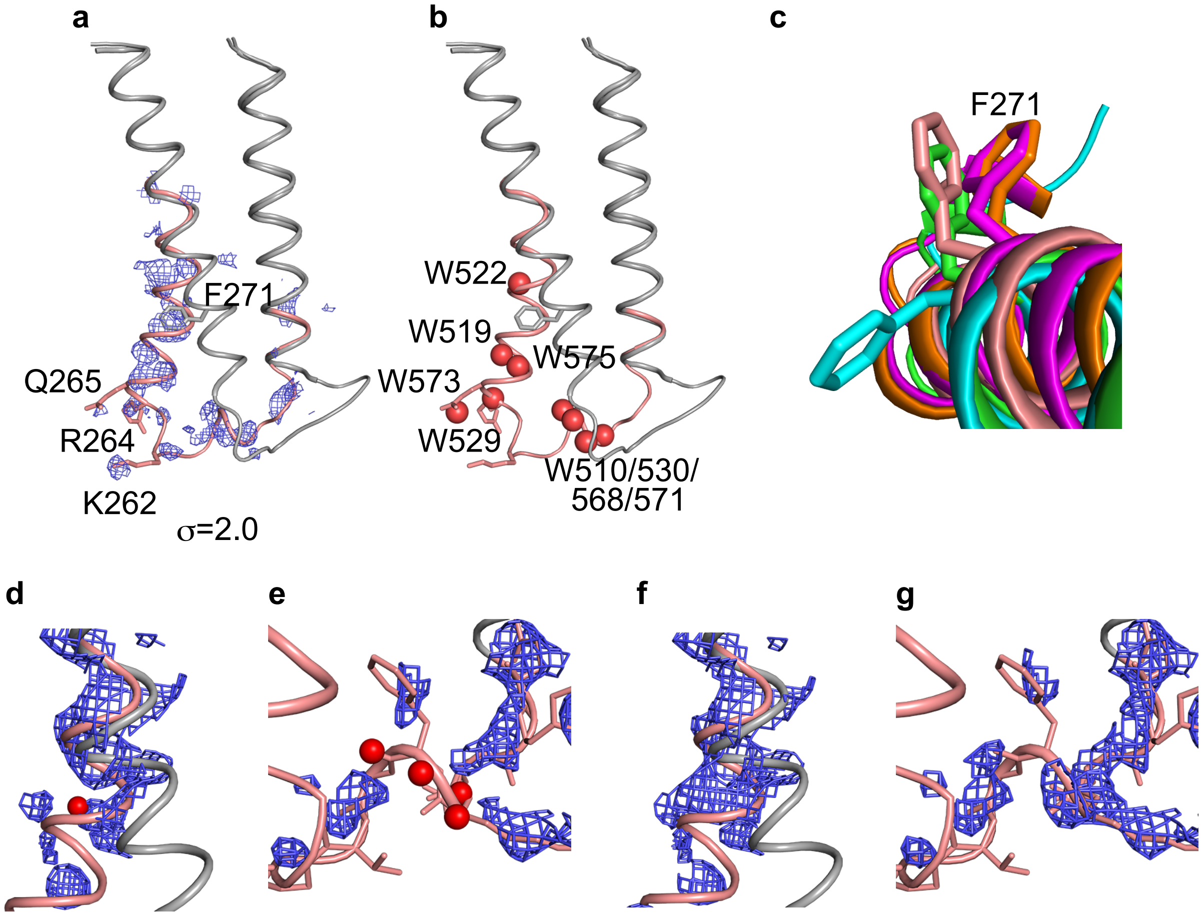

Tanabe et al. Supplementary Figure 9

Supplementary Figure 9 | The AdipoR1(D208) structure refined on the assumption of the single open-form conformation of the ICL2

a, The ***F*o–*F*c** omit maps refined on the assumption of the single open-form conformation (PDB ID 5LXG) on residues 251–280 in the closed-form structure, refined on the assumption of the dual closed-open conformations (PDB ID 6KS0). The salmon cartoon of residues 254–279 (from the middle of helix IV, through ICL2, to the middle of helix V) is shown together with the gray cartoon of the remaining parts of helices IV and V (residues 230–253 and 280–290, respectively) in the closed form (PDB ID 6KS0), and the gray cartoon of residues 230–290 in the open form (PDB ID 5LXG). The *F*o–*F*c map was prepared with autoBuster and contoured at 2.0 σ for AdipoR1(D208) in the single open-form structure (PDB ID 5LXG), and electron densities were observed (blue mesh) corresponding to the dual conformational regions in the closed form. **b**, In the single open-form structure of AdipoR1(D208) (PDB ID 5LXG), nine water molecules and the side chain of Phe271 were modeled in some electron densities observed in **a**. In contrast, there are no such electron densities in our present structure of the open form (molecule C) of AdipoR1 (A208), as shown unambiguously in Fig. 1**l**. **c**, The Phe271 side chain in the single open-form structure (PDB ID 5LXG) (cyan) is rotated by about 135˚, as compared with those in the open-form structure of molecule C of AdipoR1(A208) (salmon), the open-form structure in the present dual closed-open structure of AdipoR1(D208) (green), and the closed-form structures of molecules A (orange) and B (purple) of AdipoR1(A208). Thus, part of the electron densities corresponding to the closed form was interpreted as this side chain in the single open-form structure (PDB ID 5LXG). **d**–**g**, The *F*o–*F*c maps with (**d**, **e**) and without (**f**, **g**) the nine water molecules, contoured at 2.0 σ, were prepared for AdipoR1(D208) in the single open-form structure (PDB ID 5LXG) with autoBuster, and electron densities were observed (blue mesh). Thus, quite limited parts of the electron densities corresponding to the closed form could be interpreted as these water molecules in the single open-form structure (PDB ID 5LXG).

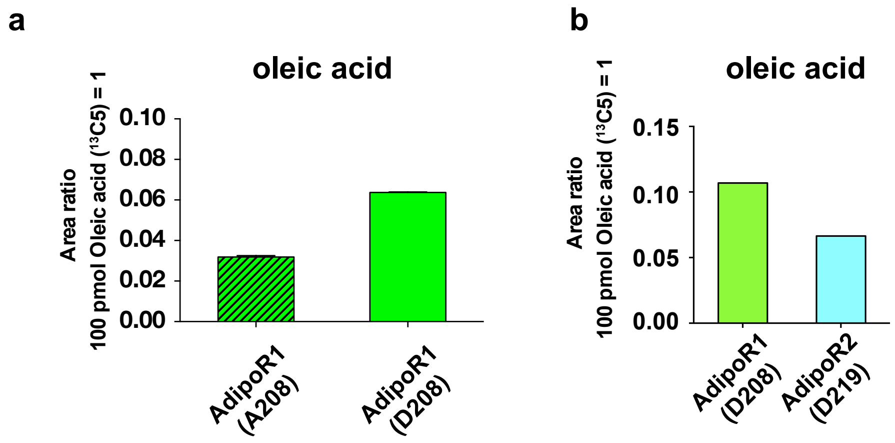

Supplementary Figure 10 | Mass spectrometric analyses of the free fatty acids in AdipoR1 (A208), AdipoR1(D208), and AdipoR2(D219)

a, Mass spectrometric analyses of the free fatty acids in the samples of AdipoR1(A208) and AdipoR1(D208) prepared from HEK293F cells. Oleic acid (18:1 free fatty acid) was detected in both of the samples. **b,** Oleic acid was also detected in the samples of AdipoR1(D208) and AdipoR2(D219) prepared from High Five cells. The amounts of oleic acid are shown as the area ratios to 100 pmol ^{13}C-labeled oleic acid standard per µg protein (or per 16.7 pmol protein). The values are presented as mean ± S.D. (n = 2) (**a, b**).

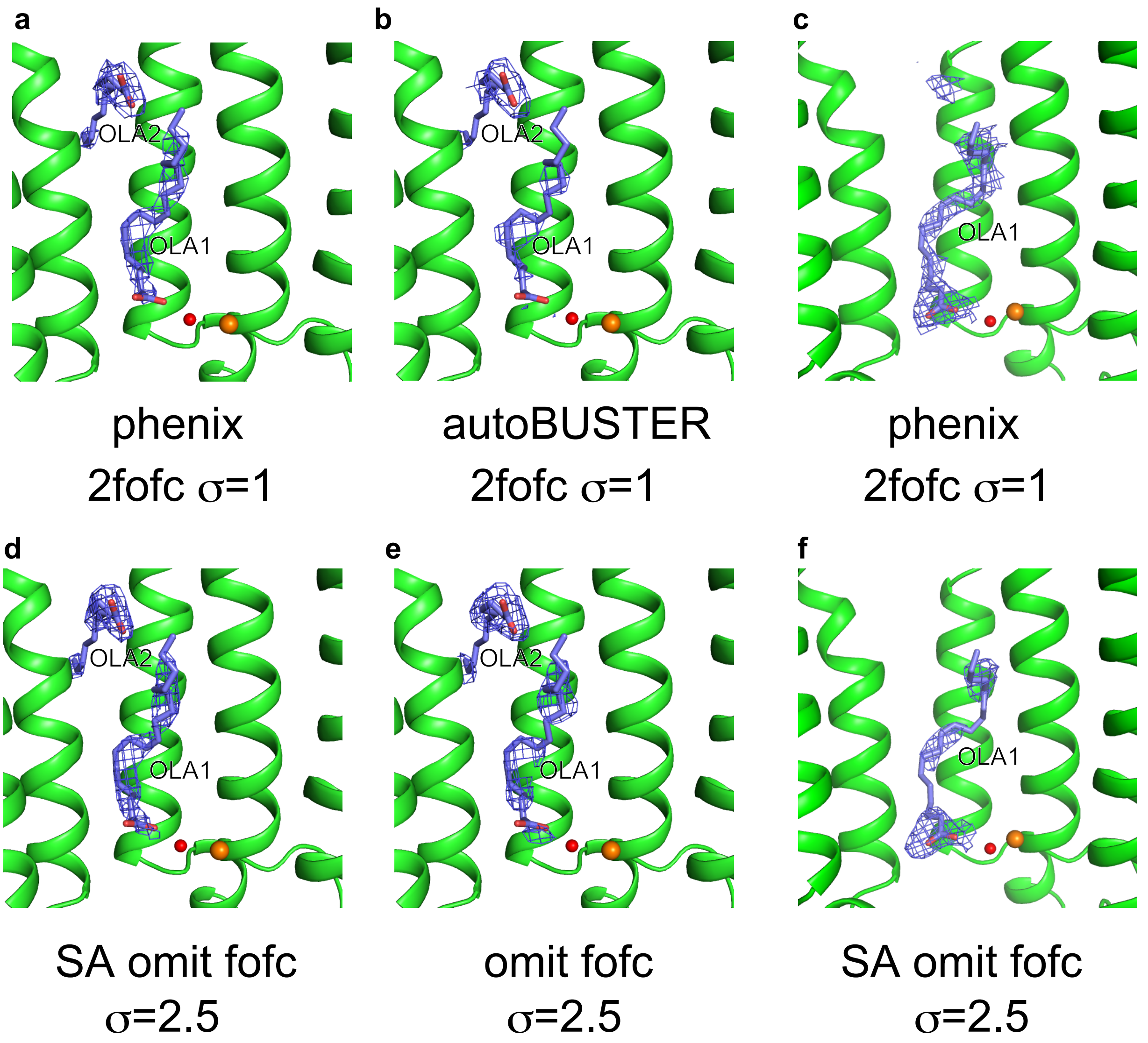

Supplementary Figure 11 | Electron density maps of the lipids in AdipoR1(A208) and AdipoR2

a, **b**, The 2*F*o–*F*c maps of two oleic acid molecules (OLA1 and OLA2) in molecule A (the closed form) of AdipoR1(A208), contoured at 1.0 σ with Phenix (**a**) and autoBUSTER (**b**). The part of OLA2 outside the major cavity is disordered (**a**, **b**). c, The 2*F*o–*F*c map of an oleic acid molecule (OLA) in AdipoR2(D219) in the closed form, contoured at 1.0 σ by Phenix. **d**, The simulated annealing (SA) *F*o–*F*c omit map of OLA1 and OLA2 in molecule A (the closed form) of AdipoR1 (A208), contoured at 2.5 σ with Phenix. **e**, The *F*o–*F*c omit map of OLA1 and OLA2 in molecule A (the closed form) of AdipoR1(A208), contoured at 2.5 σ with autoBUSTER. **f**, The SA *F*o–*F*c omit map of OLA in AdipoR2(D219) in the closed form, contoured at 2.5 σ with Phenix. In the AdipoR2(D219) structure reanalyzed by Vasiliauskaité-Brooks et al. (2017)[13] (PDB ID 5LWY), a monoolein molecule is modeled on the outer leaflet side in the major cavity, but the corresponding electron density is not observed in the present omit map. The electron densities of OLA1 were fully observed, whereas those of OLA2 were observed for half of the molecule within the major cavity and the remaining parts were disordered (**d**, **e**). Helices II and III are omitted for clarity.

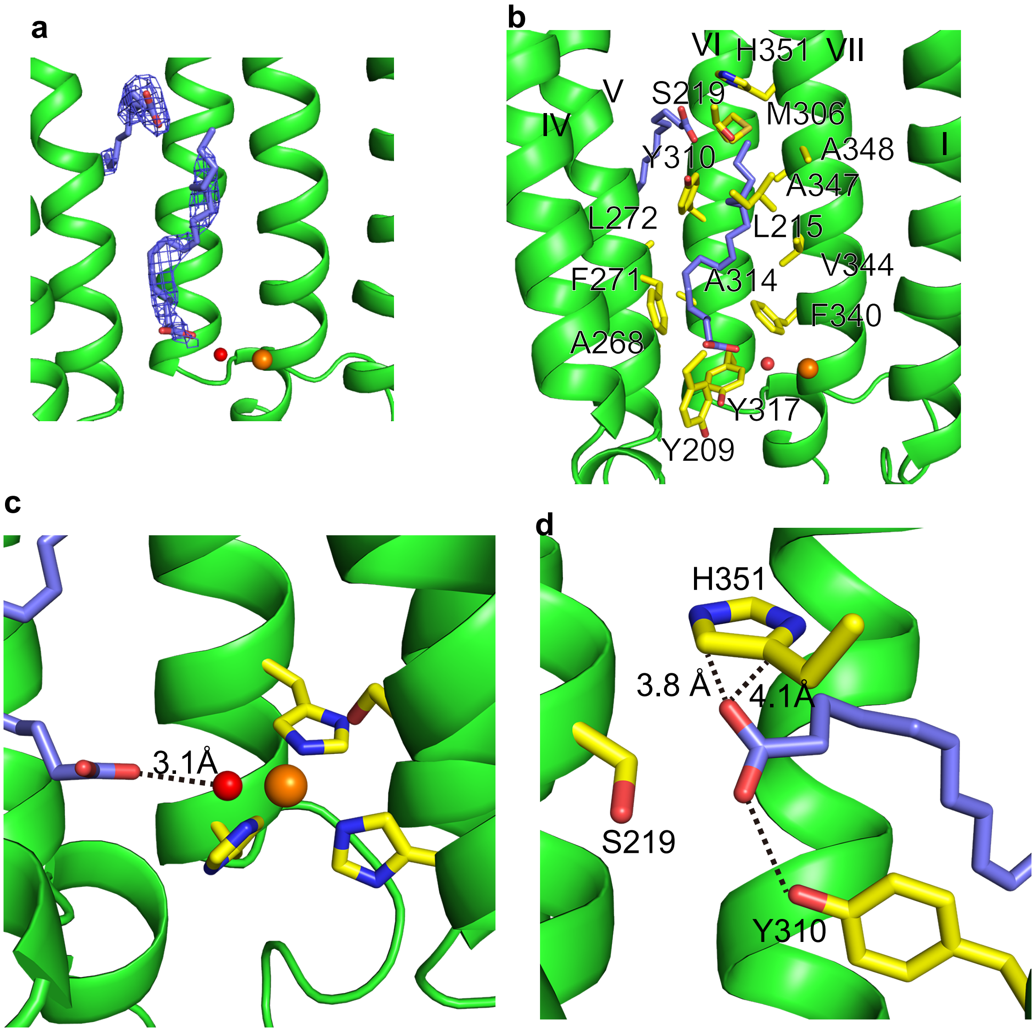

Supplementary Figure 12 | Interactions of the oleic acid molecules with AdipoR1(A208) in the closed form

a, The SA *Fo*–*F*c omit map of the two oleic acid molecules, OLA1 and OLA2 (slate blue stick models with the oxygen atoms in red), in molecule A in the closed form (green cartoon with the zinc ion and the intervening water molecule in orange and red, respectively) of AdipoR1(A208), contoured at 2.5 σ with phenix.refine. Helices II and III are omitted here for clarity. **b**, The interactions of molecule A of AdipoR1 (A208) with OLA1, depicted in the same manner as in a, except for the side chains of the OLA1-interacting residues (yellow stick models). **c**, Close-up view of the interaction of the carboxyl group of OLA1 with the water molecule bound to the zinc ion coordinated with His191, His337, and His341 in molecule A of AdipoR1(A208), depicted in the same manner as in **b**. **d**, Close-up view of the interactions of the carboxyl group of OLA2 with Ser219, Tyr310, and His351 of AdipoR1(A208) in molecule A, depicted in the same manner as in b, except that the hydrogen bonding and electrostatic interactions are indicated with dashed lines and the interatomic distances. All six of the residues shown in **c** and **d** are conserved in the adiponectin receptors.

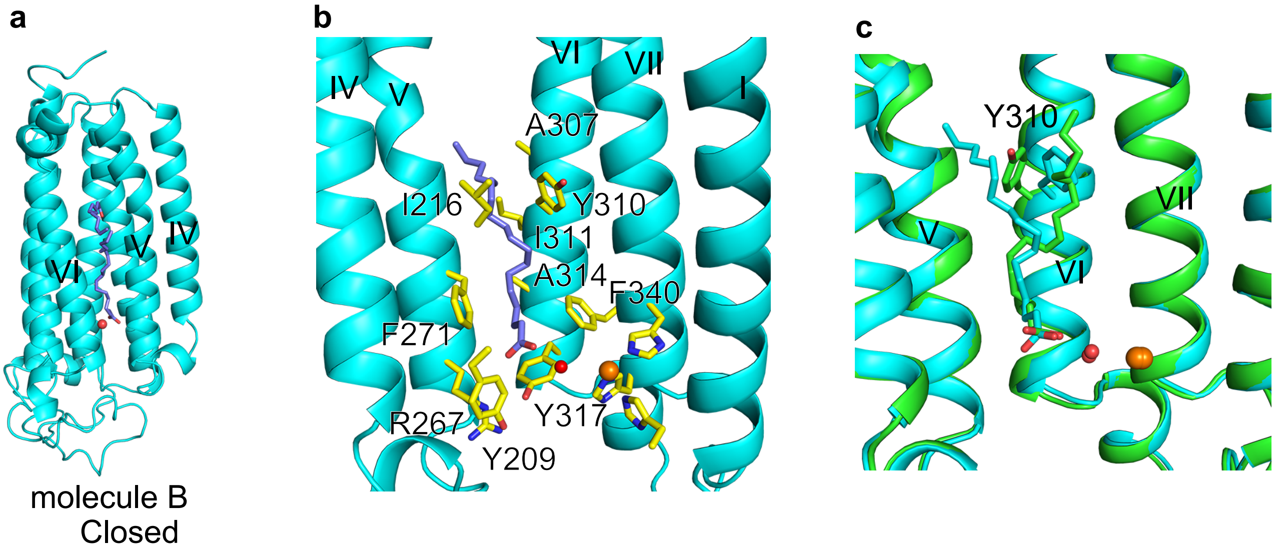

Supplementary Figure 13 | The closed-form structures of AdipoR1(A208)

a, A model of an oleic acid molecule (OLA, slate blue stick model) built in the structure of molecule B of AdipoR1(A208), viewed parallel to the membrane. The zinc ion is shown as an orange sphere. **b**, The interactions of the modeled OLA with molecule B of AdipoR1(A208). The side chains of the OLA-interacting residues are shown as yellow sticks. Helices II and III are omitted for clarity. **c**, Superimposition of helices V, VI, and VII of molecules A and B of AdipoR1 (A208). The hydrocarbon chains of OLA1 (molecule A) and OLA (molecule B), shown as stick models, are distinct because of the different positions of Tyr310 between the two molecules of AdipoR1(A208).

Supplementary Table 1 | Hydrogen bond geometry in α-helices of AdipoR1/R2

AdipoR1(A208) mol. A, helix V										
	H-Bond		O···N	C=O···N	O···H–N	H-Bond		O···N	C=O···N	O···H–N
	i	*i*+3	(Å)	(deg)	(deg)	*i*	*i*+4	(Å)	(deg)	(deg)
CT	H284	I287	3.3	111	115	H284	A288	2.8	164	151
CT	M283	T286	3.2	103	120	M283	I287	3.0	157	159
CT	T282	F285	3.6	100	109	T282	T286	3.2	143	139
CT	P281	H284	3.4	93	107	P281	F285	3.0	146	165
M2	V280	M283	3.3	112	115	V280	H284	3.0	158	158
M2	V279	T282	3.3	103	117	V279	M283	3.1	155	160
M2	G278	P281	3.9	98	-	G278	T282	3.2	141	141
M2	S277	V280	3.4	128	151	S277	P281	4.4	144	-
M2	L276	V279	3.3	115	164	L276	V280	4.7	140	139
M2	G275	G278	3.2	119	145	G275	V279	4.4	140	116
M2	L274	S277	3.5	103	149	L274	G278	4.5	132	138
M2	G273	L276	3.2	96	115	G273	S277	3.4	144	125
M1	L272	G275	3.1	110	117	L272	L276	2.9	153	142
M1	F271	L274	3.7	84	120	F271	G275	3.5	129	150
M1	V270	G273	3.2	109	106	V270	L274	2.9	154	140
M1	G269	L272	3.6	92	116	G269	G273	2.8	143	158
M1	A268	F271	3.7	112	105	A268	L272	3.0	156	142
NT	R267	V270	3.7	94	125	R267	F271	3.6	141	176
NT	T266	G269	3.1	111	110	T266	V270	3.0	163	148
NT	Q265	A268	3.3	98	117	Q265	G269	3.0	150	158
NT	R264	R267	3.5	102	107	R264	A268	3.2	145	146

AdipoR1(A208) mol. B, helix V										
	H-Bond		O···N	C=O···N	O···H–N	H-Bond		O···N	C=O···N	O···H–N
	i	*i*+3	(Å)	(deg)	(deg)	*i*	*i*+4	(Å)	(deg)	(deg)
CT	H284	I287	3.4	101	117	H284	A288	2.9	154	163
CT	M283	T286	3.1	102	112	M283	I287	2.7	159	150
CT	T282	F285	3.4	102	110	T282	T286	3.0	147	145
CT	P281	H284	3.4	98	106	P281	F285	2.9	150	165
M2	V280	M283	3.2	112	113	V280	H284	2.9	160	161
M2	V279	T282	3.8	89	120	V279	M283	3.6	133	157
M2	G278	P281	3.7	95	-	G278	T282	3.1	143	146
M2	S277	V280	4.2	112	157	S277	P281	4.7	131	-
M2	L276	V279	3.4	109	165	L276	V280	5.0	137	137
M2	G275	G278	3.5	122	171	G275	V279	4.9	130	109
M2	L274	S277	3.5	107	159	L274	G278	5.2	128	129
M2	G273	L276	3.0	102	124	G273	S277	3.6	146	107
M1	L272	G275	2.9	113	118	L272	L276	3.0	153	132
M1	F271	L274	3.5	90	110	F271	G275	3.1	137	145
M1	V270	G273	3.4	106	101	V270	L274	2.9	153	149
M1	G269	L272	3.4	97	116	G269	G273	3.0	149	175
M1	A268	F271	3.6	102	107	A268	L272	2.9	149	142
NT	R267	V270	3.3	107	118	R267	F271	3.2	157	174
NT	T266	G269	3.2	104	113	T266	V270	2.9	158	152
NT	Q265	A268	3.4	101	107	Q265	G269	2.9	150	148
NT	R264	R267	3.4	106	119	R264	A268	3.4	149	165

AdipoR1(A208) mol. C, helix V										
	H-Bond		O···N	C=O···N	O···H–N	H-Bond		O···N	C=O···N	O···H–N
	i	*i*+3	(Å)	(deg)	(deg)	*i*	*i*+4	(Å)	(deg)	(deg)
CT	H284	I287	3.3	100	124	H284	A288	3.2	154	163
CT	M283	T286	3.1	99	111	M283	I287	2.8	155	142
CT	T282	F285	3.4	102	108	T282	T286	2.9	147	144
CT	P281	H284	3.6	90	105	P281	F285	3.0	140	160
M2	V280	M283	3.5	112	116	V280	H284	3.2	154	161
M2	V279	T282	3.4	97	128	V279	M283	3.4	146	165
M2	G278	P281	3.7	99	-	G278	T282	3.1	145	129
M2	S277	V280	4.8	80	138	S277	P281	4.7	114	-
M2	L276	V279	3.8	98	160	L276	V280	4.7	131	152
M2	G275	G278	3.7	117	145	G275	V279	4.9	131	126
M2	L274	S277	3.5	98	155	L274	G278	4.8	119	103
M2	G273	L276	3.7	89	109	G273	S277	3.1	135	102
M1	L272	G275	3.2	105	112	L272	L276	2.8	159	158
M1	F271	L274	3.5	94	122	F271	G275	3.2	140	152
M1	V270	G273	3.4	97	106	V270	L274	2.9	147	141
M1	G269	L272	3.4	97	114	G269	G273	2.8	149	156
M1	A268	F271	3.7	93	109	A268	L272	3.0	139	141
NT	R267	V270	3.8	95	117	R267	F271	3.4	139	158
NT	T266	G269	3.1	102	117	T266	V270	2.7	159	163
NT	Q265	A268	3.6	96	106	Q265	G269	2.7	144	125
NT	R264	R267	3.6	97	105	R264	A268	3.0	146	160

Supplementary Table 1 (continued)

AdipoR1(A208) mol. A, helix III									
H-Bond		O···N	C=O···N	O···H−N	H-Bond		O···N	C=O···N	O···H−N
i	*i*+3	(Å)	(deg)	(deg)	*i*	*i*+4	(Å)	(deg)	(deg)
W223	Y226	3.6	110	118	W223	S227	3.0	158	153
P222	Y225	3.3	100	127	P222	Y226	3.4	150	165
V221	L224	3.9	97	108	V221	Y225	3.2	141	132
F220	W223	3.3	103	128	F220	L224	2.7	154	163
S219	P222	4.0	109	-	S219	W223	3.7	144	157
G218	V221	3.6	99	140	G218	P222	4.3	141	-
M217	F220	3.2	118	135	M217	V221	3.7	151	134
I216	S219	3.3	101	131	I216	F220	3.5	148	143
L215	G218	3.3	102	101	L215	S219	2.8	152	126
L214	M217	3.6	95	103	L214	G218	2.8	145	158
A213	L215	3.3	107	108	A213	M217	2.8	161	169
I212	L214	3.2	106	119	I212	I216	3.1	156	162
G211	A213	3.3	101	106	G211	L215	3.0	152	148

AdipoR1(A208) mol. B, helix III									
H-Bond		O···N	C=O···N	O···H−N	H-Bond		O···N	C=O···N	O···H−N
i	*i*+3	(Å)	(deg)	(deg)	*i*	*i*+4	(Å)	(deg)	(deg)
W223	Y226	3.3	113	121	W223	S227	2.8	166	161
P222	Y225	3.7	90	124	P222	Y226	3.5	136	155
V221	L224	3.9	101	108	V221	Y225	3.4	144	140
F220	W223	3.5	96	117	F220	L224	2.9	146	165
S219	P222	4.3	98	-	S219	W223	3.9	132	142
G218	V221	3.6	89	146	G218	P222	4.3	139	-
M217	F220	3.2	117	143	M217	V221	3.7	152	129
I216	S219	3.1	107	135	I216	F220	3.4	153	133
L215	G218	3.8	94	104	L215	S219	3.2	139	123
L214	M217	3.7	92	102	L214	G218	2.9	142	157
A213	L215	3.0	111	113	A213	M217	2.7	168	174
I212	L214	3.4	100	116	I212	I216	3.1	147	150
G211	A213	3.2	104	103	G211	L215	2.8	151	157

AdipoR1(A208) mol. C, helix III									
H-Bond		O···N	C=O···N	O···H−N	H-Bond		O···N	C=O···N	O···H−N
i	*i*+3	(Å)	(deg)	(deg)	*i*	*i*+4	(Å)	(deg)	(deg)
W223	Y226	3.5	110	119	W223	S227	2.9	162	160
P222	Y225	4.1	81	126	P222	Y226	3.6	124	154
V221	L224	3.7	106	104	V221	Y225	3.2	152	141
F220	W223	3.4	104	126	F220	L224	2.9	156	170
S219	P222	4.4	96	-	S219	W223	3.9	131	136
G218	V221	4.0	91	137	G218	P222	4.4	132	-
M217	F220	3.0	119	142	M217	V221	3.4	164	142
I216	S219	3.4	101	133	I216	F220	3.4	146	128
L215	G218	3.7	91	101	L215	S219	3.1	138	129
L214	M217	3.6	94	106	L214	G218	2.9	144	158
A213	L215	3.2	101	114	A213	M217	2.8	156	167
I212	L214	3.0	115	120	I212	I216	3.0	158	148
G211	A213	3.3	100	112	G211	L215	3.1	149	146

Supplementary Table 1 (continued)

AdipoR1(D208)_dual_closed, helix V										
	H-Bond		O···N	C=O···N	O···H–N	H-Bond		O···N	C=O···N	O···H–N
	i	*i*+3	(Å)	(deg)	(deg)	*i*	*i*+4	(Å)	(deg)	(deg)
CT	H284	I287	3.4	103	139	H284	A288	2.8	156	160
CT	M283	T286	3.2	99	108	M283	I287	2.9	151	124
CT	T282	F285	3.3	104	112	T282	T286	2.9	151	152
CT	P281	H284	3.4	93	103	P281	F285	2.9	146	155
M2	V280	M283	3.5	108	110	V280	H284	3.0	153	156
M2	V279	T282	3.4	99	114	V279	M283	3.1	151	171
M2	G278	P281	3.8	101	-	G278	T282	3.2	145	143
M2	S277	V280	3.7	113	151	S277	P281	4.5	137	-
M2	L276	V279	3.1	115	164	L276	V280	4.6	140	137
M2	G275	G278	3.2	123	157	G275	V279	4.5	136	117
M2	L274	S277	3.3	115	154	L274	G278	4.6	138	132
M2	G273	L276	3.6	94	125	G273	S277	3.9	135	124
M1	L272	G275	3.2	108	109	L272	L276	2.9	161	144
M1	F271	L274	3.4	98	115	F271	G275	2.9	148	156
M1	V270	G273	3.1	108	109	V270	L274	3.0	153	150
M1	G269	L272	3.3	96	106	G269	G273	2.7	150	157
M1	A268	F271	3.7	108	103	A268	L272	2.9	151	144
NT	R267	V270	3.4	108	127	R267	F271	3.6	155	171
NT	T266	G269	3.2	102	116	T266	V270	3.0	156	148
NT	Q265	A268	3.5	104	108	Q265	G269	3.1	147	141
NT	R264	R267	3.1	74	119	R264	A268	3.3	154	163

AdipoR1(D208)_dual_open, helix V										
	H-Bond		O···N	C=O···N	O···H–N	H-Bond		O···N	C=O···N	O···H–N
	i	*i*+3	(Å)	(deg)	(deg)	*i*	*i*+4	(Å)	(deg)	(deg)
CT	H284	I287	3.4	103	139	H284	A288	2.8	156	160
CT	M283	T286	3.2	99	108	M283	I287	2.9	151	124
CT	T282	F285	3.3	104	112	T282	T286	2.9	151	152
CT	P281	H284	3.4	93	103	P281	F285	2.9	146	155
M2	V280	M283	3.5	110	110	V280	H284	3.0	154	156
M2	V279	T282	3.5	103	118	V279	M283	3.2	154	174
M2	G278	P281	4.0	95	-	G278	T282	3.4	138	144
M2	S277	V280	4.1	101	136	S277	P281	4.5	135	-
M2	L276	V279	3.3	118	168	L276	V280	4.5	147	147
M2	G275	G278	3.8	117	172	G275	V279	5.2	127	126
M2	L274	S277	3.4	104	149	L274	G278	4.6	134	119
M2	G273	L276	4.0	93	105	G273	S277	3.2	137	111
M1	L272	G275	3.3	104	109	L272	L276	2.9	158	168
M1	F271	L274	3.3	95	112	F271	G275	2.9	148	158
M1	V270	G273	3.3	89	105	V270	L274	2.9	151	147
M1	G269	L272	3.2	100	116	G269	G273	2.9	164	166
M1	A268	F271	3.6	85	103	A268	L272	2.8	148	137
NT	R267	V270	3.8	101	119	R267	F271	3.5	144	168
NT	T266	G269	3.1	94	115	T266	V270	3.0	149	165
NT	Q265	A268	3.1	110	113	Q265	G269	2.9	149	134
NT	R264	R267	3.3	92	108	R264	A268	3.1	143	157

AdipoR1(D208)_single_Open, helix V										
	H-Bond		O···N	C=O···N	O···H–N	H-Bond		O···N	C=O···N	O···H–N
	i	*i*+3	(Å)	(deg)	(deg)	*i*	*i*+4	(Å)	(deg)	(deg)
CT	H284	I287	3.5	104	114	H284	A288	3.2	154	168
CT	M283	T286	3.0	110	109	M283	I287	2.9	163	159
CT	T282	F285	3.3	104	116	T282	T286	3.3	146	155
CT	P281	H284	3.3	99	107	P281	F285	2.9	149	152
M2	V280	M283	3.6	110	111	V280	H284	3.0	156	152
M2	V279	T282	3.5	106	107	V279	M283	3.1	158	171
M2	G278	P281	3.6	107	-	G278	T282	3.2	154	160
M2	S277	V280	3.7	117	121	S277	P281	3.9	151	-
M2	L276	V279	3.2	117	145	L276	V280	4.1	154	156
M2	G275	G278	3.5	109	131	G275	V279	4.1	142	133
M2	L274	S277	3.7	98	120	L274	G278	3.7	139	148
M2	G273	L276	3.5	102	105	G273	S277	2.8	154	147
M1	L272	G275	3.5	118	109	L272	L276	3.0	166	162
M1	F271	L274	3.2	116	124	F271	G275	3.5	160	166
M1	V270	G273	3.0	106	114	V270	L274	3.3	149	153
M1	G269	L272	3.2	102	108	G269	G273	3.0	150	145
M1	A268	F271	3.2	106	104	A268	L272	2.7	156	151
NT	R267	V270	3.2	113	115	R267	F271	3.0	161	153
NT	T266	G269	2.6	120	118	T266	V270	3.1	168	156
NT	Q265	A268	3.0	126	130	Q265	G269	3.7	139	141
NT	R264	R267	5.3	95	157	R264	A268	6.8	91	167

AdipoR2(D219), helix V										
	H-Bond		O···N	C=O···N	O···H–N	H-Bond		O···N	C=O···N	O···H–N
	i	*i*+3	(Å)	(deg)	(deg)	*i*	*i*+4	(Å)	(deg)	(deg)
CT	H295	I298	3.5	100	117	H295	S299	3.3	148	156
CT	L294	V297	3.4	102	114	L294	I298	3.0	153	155
CT	T293	Y296	3.1	105	117	T293	V297	2.9	156	154
CT	P292	H295	3.5	96	104	P292	Y296	3.0	145	146
M2	I291	L294	3.5	105	110	I291	H295	3.1	153	162
M2	I290	T293	3.4	96	114	I290	L294	2.9	148	163
M2	G289	P292	3.6	102	-	G289	T293	3.1	148	147
M2	S288	I291	3.3	129	166	S288	P292	4.5	136	-
M2	L287	I290	3.2	114	162	L287	I291	5.1	130	132
M2	G286	G289	3.3	120	160	G286	I290	5.0	127	116
M2	L285	S288	3.4	88	143	L285	G289	4.5	132	130
M2	G284	L287	3.8	88	103	G284	S288	3.0	134	115
M1	L283	G286	3.3	113	107	L283	L287	2.8	159	165
M1	F282	L285	3.2	99	121	F282	G286	3.2	150	157
M1	V281	G284	3.5	101	108	V281	L285	3.0	146	134
M1	G280	L283	3.5	94	106	G280	G284	2.7	106	156
M1	A279	F282	3.6	109	109	A279	L283	2.9	155	151
NT	R278	V281	3.6	97	118	R278	F282	3.3	146	174
NT	V277	G280	3.4	106	110	V277	V281	3.0	155	150
NT	G276	A279	3.5	93	114	G276	G280	3.2	143	163
NT	R275	R278	3.7	92	109	R275	A279	3.2	136	144

Supplementary Table 1 | Hydrogen bonding geometries of helices in AdipoR1 and AdipoR2. The hydrogen bonding geometries of residue *i* with residue *i*+3 (left) and with residue *i*+4 (right) in helix V (**a**–**c**) and helix III (**d**–**f**) of molecules A (**a**, **d**), B (**b**, **e**), and C (**c**, **f**) of AdipoR1 (A208) (6KRZ) and in helix V in the closed form (**g**) and the open form (**h**) of AdipoR1 (D208), based on the present dual closed-open conformational assumption (6KS0), AdipoR1 (D208) on the single open-only conformational assumption (5XLG) (**i**), and AdipoR2 (D219) (6KS1). For helix V of AdipoR1, the NT, M1, M2, and CT regions are indicated.

O···N: the distances (Å) from the main-chain carbonyl oxygen atom of residue *i* to the main-chain nitrogen atom of residue *i*+3 (left) or residue *i*+4 (right). The O···N distances in the ranges of 2.6–3.2 Å and 3.3–3.5 Å, highlighted in cyan and green, respectively, are required for strong and weak hydrogen bond formation, respectively, while those in the range of 3.6–5.3 Å, indicating no hydrogen bonds, are highlighted in gray. The only exceptional case of 6.8 Å (dark gray) in AdipoR1 (D208), based on the single open-only conformational assumption (5XLG), may indicate no helix formation.

C=O···N: the angle (°) formed by the main-chain carbonyl carbon and oxygen atoms of residue *i* and the main-chain nitrogen atom of residue *i*+3 (left) or residue *i*+4 (right). The C=O···N angles in the range of 90–180°, highlighted in cyan, are required for hydrogen bonding, while those outside of this range, indicating no hydrogen bond formation, are highlighted in gray.

O···H–N: the angle (°) formed by the main-chain carbonyl oxygen atom of residue *i* and the main-chain amide hydrogen and nitrogen atoms of residue *i*+3 (left) or residue *i*+4 (right), except for a Pro residue at position *i*+3 or *i*+4 (-). The O···H–N angles in the range of 130–180°, highlighted in cyan, are required for hydrogen bonding, while those outside of this range, indicating no hydrogen bond formation, are highlighted in gray.

H-Bond: If all three parameters, the O···N distance and the C=O···N and O···H–N angles, are in the ranges required for hydrogen bonding, as defined above, then the residue pairs (*i*, *i*+3) or (*i*, *i*+4) are concluded to form the main-chain C=O···H–N hydrogen bond, and are highlighted in cyan (strong hydrogen bond) and light green (weak hydrogen bond).

The hydrogen bonding geometries of residue *i* with residue *i*+3 (left) and with residue *i*+4 (right) in helix V (**a**–**c**) and helix III (**d**–**f**) of molecules A (**a**, **d**), B (**b**, **e**), and C (**c**, **f**) of AdipoR1 (A208) (6KRZ) and in helix V in the closed form (**g**) and the open form (**h**) of AdipoR1 (D208), based on the present dual closed-open conformational assumption (6KS0), AdipoR1 (D208) on the single

open-only conformational assumption (5XLG) (**i**), and AdipoR2 (D219) (6KS1). For helix V of AdipoR1, the NT, M1, M2, and CT regions are indicated.

Supplementary Table 2 | The (ϕ, ψ) angles of helix V of AdipoR1 A208

		mol.A		mol.B		mol.C	
		ϕ (˚)	ψ (˚)	ϕ (˚)	ψ (˚)	ϕ (˚)	ψ (˚)
273	G	-63.4	-27.7	-60.9	-31.7	-65.6	-28
274	L	-66.4	-38.3	-62.2	-35.9	-72.3	-38.3
275	G	-76.2	-25.3	-74.4	-22.7	-72.7	-38.3
276	L	-64.2	-20.5	-73.2	1.7	-83	8.7
277	S	-64.6	-32.1	-65.7	-27.2	-82.8	6.9
278	G	-68.4	-3.3	-88.4	7.8	-110.1	-13.5
279	V	-71.1	-36.4	-72.8	-30.4	-71.6	-33.9
280	V	-66	-52.1	-83.3	-44.9	-72.3	-49.3
281	P	-65.8	-24	-60.3	-30.3	-68.5	-17.1
3_{10} helix		-49	-26				
α helix		-57	-47				

Supplementary Notes

Oleic acid molecules in the crystal structures of AdipoR1(A208)

In this study, we performed mass spectrometric analyses[1] of the free fatty acids in the AdipoR1(A208) and AdipoR1(D208) samples used for crystallization in the present and previous studies, respectively (Supplementary Figure 10). In both samples, oleic acid was predominantly detected. The molar ratios of oleic acid to AdipoR1(A208) and AdipoR1(D208) are 0.15 and 0.28, respectively. Other free fatty acids were below the detection level.

In the AdipoR1(A208) structure (PDB ID 6KRZ), among the long extra electron densities within the internal cavities of the three AdipoR1 molecules, those in molecule A are particularly strong (Supplementary Figures 11, 12a). According to our mass spectrometric analyses, the long extra electron densities in molecule A of AdipoR1(A208), designated hereafter as AdipoR1–6KRZ-A, were interpreted as oleic acid molecules (Fig. 1a). In more detail, two oleic acid molecules, OLA1 and OLA2, were modeled in the long extra electron densities on the inner and outer leaflet sides, respectively, of the closed-form cavity in AdipoR1–6KRZ-A (Supplementary Figure 12a, b). The carboxyl group of OLA1 is involved in a water-mediated interaction with the zinc ion (Supplementary Figure 12c). The hydrocarbon chain of OLA1 runs parallel to the transmembrane helices, and interacts with numerous amino-acid side chains, which are mostly hydrophobic, in helices II, III, V, VI, and VII (Supplementary Figure 12b, Table 2).

Moreover, as for the extra electron density on the outer leaflet side of the major cavity, OLA2 could be modeled up to the tiny LB opening of the major cavity in AdipoR1–6KRZ-A, and the rest of the molecule was disordered outside the protein molecule. The carboxyl group of OLA2 is observed in the middle of the major cavity (Supplementary Figure 12b). Three hydrophilic residues, Ser219, Tyr310, and His351, which are conserved in the adiponectin receptors, surround the carboxyl group

(Supplementary Figure 12d). Presumably, the carboxyl group of OLA2 hydrogen bonds with the hydroxyl group of Tyr310 and electrostatically interacts with His351.

On the other hand, the extra electron densities in molecule B were weaker than those in molecule A. Therefore, we did not include a lipid molecule in the structure of molecule B (AdipoR1–6KRZ-B). Nevertheless, the weak electron densities helped us to build docking models of the two oleic molecules, OLA1 and OLA2, in AdipoR1–6KRZ-B (Supplementary Figure 13a, b), corresponding to those in molecule A of AdipoR1(A208). Intriguingly, Tyr310 assumes a slightly different conformation in molecule B from that in molecule A, and correspondingly, the distal half of the hydrocarbon chain, from the center to the end opposite the carboxyl group, runs on the other side of Tyr310 as compared to molecule A (Supplementary Figure 13c). Some poor extra electron densities exist in the major cavity of AdipoR1(D208), but we could not reliably model any lipid molecules because of the dual conformations.

Oleic acid and/or monoolein molecules in the structures of AdipoR2(D219)

We previously observed extra electron densities within the major cavity in our crystal structure of AdipoR2(D219) (PDB ID 3WXW)[2], which is designated hereafter as AdipoR2–3WXW. Subsequently, Vasiliauskaité-Brooks *et al.*[3] also observed extra electron densities in their AdipoR2(D219) crystals, and interpreted them as an oleic acid molecule, the major fatty acid in the insect cell membrane, and a monoolein molecule (designated as AdipoR2–5LX9). The oleic acid and monoolein molecules positionally correspond to OLA1 and OLA2, respectively, in the present structure of AdipoR1–6KRZ-A. Furthermore, they reanalyzed our previous diffraction data set for AdipoR2–3WXW[2], and reported similar oleic acid and monoolein structures in the major cavity[3] (AdipoR2–5LWY).

In this study, we also confirmed that the predominant fatty acid in our AdipoR2(D219) sample is oleic acid (Supplementary Figure 10b), and therefore we reanalyzed our previous diffraction data set for AdipoR2–3WXW[2], and revised the

AdipoR2 structure with PDB ID 6KS1 (AdipoR2–6KS1). Thus, an oleic acid molecule corresponding to OLA1 in AdipoR1–6KRZ-A was identified in AdipoR2–6KS1. However, in our electron density map of AdipoR2–6KS1, the electron density corresponding to OLA2 was much weaker than that of OLA1, and also weaker than that of OLA2 in AdipoR1–6KRZ-A (Supplementary Figure 11c). Therefore, we did not model any molecule in the position corresponding to OLA2.

As for the AdipoR2–5LX9 structure by Vasiliauskaité-Brooks *et al.*[3], we do not support the model of a monoolein molecule in the electron density corresponding to OLA2, for the following reasons. The hydrophilic amino acid residues Ser219, Tyr310, and His351, which bind the carboxyl group of OLA2 in the major cavity of AdipoR1–6KRZ-A, are conserved as Ser230, Tyr321, and His362 in AdipoR2. Therefore, we may postulate the same OLA2 binding mode for AdipoR2. In contrast, in AdipoR2–5LX9, the hydrophobic end of the modeled monoolein molecule faces the three hydrophilic residues. For the same reason, it is unreasonable to model a monoolein molecule in the putative OLA2-binding site in the AdipoR2–5LWY structure[3], which was reanalyzed on the basis of our data set[2]. Moreover, the electron density is too weak to model any molecule, as described above, possibly because the LB aperture is larger in AdipoR2–6KS1 than in AdipoR1–6KRZ-A.

The OLA1-binding modes in AdipoR1–6KRZ-A, AdipoR2–5LWY, and AdipoR2–6KS1 (Supplementary Figure 11) are the same: a water molecule intervenes between the carboxyl group of OLA1 and the zinc ion. In contrast, the intervening water molecule is missing and the carboxyl group of the oleic acid molecule is directly coordinated to the zinc ion in AdipoR2–5LX9. Therefore, we reanalyzed this diffraction data set, and confirmed that the water-mediated interaction between the carboxyl group of the oleic acid molecule and the zinc ion is also possible, while another oleic acid molecule (OLA2) can be modeled instead of the monooleic molecule described above (not shown).

Discussion

The downstream signaling of AdipoR2 appears to depend on the putative hydrolytic activity, because the single Ala mutations of Asp219 and His348 from the putative catalytic site of AdipoR2 decreased the adiponectin-stimulated UCP2 expression in the PPAR-α pathway signaling[2]. Therefore, the putative hydrolytic activity of AdipoR1 could still be involved in other signaling pathways besides the AMPK activation. The closed form of AdipoR1(A208) can only fully accommodate one oleic acid molecule (OLA1) within the major cavity, which has a few tiny openings. In contrast, in the open form, the apertures of the major cavity are much larger than those in the closed form. Considering that the putative hydrolysis substrates may have two hydrocarbon chains, the open-form major cavity may be able to accommodate such substrates. Furthermore, the opening of the major cavity toward the inner leaflet of the lipid bilayer (LB opening) in the open form is expected to be large enough for the substrate to enter the major cavity. Therefore, the putative hydrolysis catalyzed by AdipoR1, as well as that by AdipoR2, is performed in the open form, rather than in the closed form. After the hydrolysis, one of the putative products may be a free fatty acid. The oleic acid (OLA1) in the closed form of AdipoR1/AdipoR2 is positioned with its carboxyl group in the proximity of the zinc ion, indicating that the structures are relevant to the binding mode of the putative product or oleic acid.

On the other hand, in the present study, we found that the second oleic acid molecule (OLA2) is bound with its carboxyl group interacting with the conserved hydrophilic residues in the middle of the major cavity of AdipoR1(A208) (Supplementary Figure 12b, d). Here, nearly half of the hydrocarbon chain of OLA2 has passed through the LB opening of the major cavity. Interestingly, the two OLA molecules in the first and second sites are bound with their carboxyl groups closer to the cytoplasm than the hydrocarbon chains. This orientation suggests that the oleic acid molecule bound in the OLA1 site can move to the OLA2 site, with nearly half of its hydrocarbon chain protruding through the LB opening of the major cavity. We further speculate that OLA2

can leave the major cavity by passing through the LB opening, when the LB opening is sufficiently enlarged for the sake of the conformational flexibility of the Gly-rich M2 region of helix V, as in the structure of molecule B of AdipoR1(A208) (Fig. 1). Correspondingly, the electron densities corresponding to OLA1 and OLA2 are weaker in molecule B than in molecule A. In contrast, the opposite movement; *i.e.*, entering the major cavity through the LB opening, is quite unlikely for the negatively-charged carboxyl group of oleic acid. Thus, the present structure of molecule A with two oleic acids may provide a working hypothesis for understanding how the product fatty acid exits the catalytic, major cavity.

As for the substrate specificity of the putative hydrolytic activities of the adiponectin receptors, it was recently reported that AdipoR2 possesses ceramidase activity, with K_m and k_{cat} values of 15.6 μM and $0.49 \times 10^{-3}\ s^{-1}$ [3]. In contrast, Wang *et al.* previously reported that the adiponectin-dependent ceramidase activity is ascribed, not to the adiponectin receptor 1 (AdipoR1) itself, but to another single transmembrane protein, neutral ceramidase (nCDase), in the downstream reaction[4]. Furthermore, AdipoR1 and nCDase reportedly form a signaling complex together with caveolin-1 (Cav1)[4]. The K_m and k_{cat} values of nCDase were 33.41 μM and 1.032 sec^{-1}, as reported by Airola *et al.*[5], representing a nearly 1,000-fold faster reaction than the above-mentioned ceramidase activity of AdipoR2. Thus, the possibility that the adiponectin receptors also have another lipid hydrolase activity cannot be excluded[6]. Therefore, the present structures and the oleic acid-binding modes of the adiponectin receptors in the closed and/or open forms provide important information for future studies on the catalytic activities of the adiponectin receptors.

Supplementary References

1 Okudaira, M. *et al.* Separation and quantification of 2-acyl-1-lysophospholipids and 1-acyl-2-lysophospholipids in biological samples by LC-MS/MS. *J. Lipid Res.* **55**, 2178–92 (2014).

2 Tanabe, H. *et al.* Crystal structures of the human adiponectin receptors. *Nature* **520**, 312–316 (2015).

3 Vasiliauskaité-Brooks, I. *et al.* Structural insights into adiponectin receptors suggest ceramidase activity. *Nature* **544**, 120–123 (2017).

4 Wang, Y. *et al.* Adiponectin inhibits tumor necrosis factor-α-induced vascular inflammatory response via caveolin-mediated ceramidase recruitment and activation. *Circ. Res.* **114**, 792–805 (2014).

5 Airola, M. V. *et al.* Structural Basis for Ceramide Recognition and Hydrolysis by Human Neutral Ceramidase. *Structure* **23**, 1482–1491 (2015).

6 Holland, W. L. & Scherer, P. E. Structural biology: Receptors grease the metabolic wheels. *Nature* **544**, 42–44 (2017).
