# Supplementary material for: Human adiponectin receptor AdipoR1 assumes closed and open structures
Source: Commun Biol. 2020 Aug 14;3:446. doi: 10.1038/s42003-020-01160-4 (PMC7427782; doi:10.1038/s42003-020-01160-4)
Supplement: Supplementary file 2 — Reporting Summary [file 42003_2020_1160_MOESM2_ESM.pdf]

nature research

Corresponding author(s): COMMSBIO-18-1143A

Last updated by author(s): May 19, 2020

Reporting Summary

Nature Research wishes to improve the reproducibility of the work that we publish. This form provides structure for consistency and transparency in reporting. For further information on Nature Research policies, see Authors & Referees and the Editorial Policy Checklist.

Statistics

For all statistical analyses, confirm that the following items are present in the figure legend, table legend, main text, or Methods section.

n/a	Confirmed	
☐	☒	The exact sample size (*n*) for each experimental group/condition, given as a discrete number and unit of measurement
☒	☐	A statement on whether measurements were taken from distinct samples or whether the same sample was measured repeatedly
☐	☒	The statistical test(s) used AND whether they are one- or two-sided<br>*Only common tests should be described solely by name; describe more complex techniques in the Methods section.*
☒	☐	A description of all covariates tested
☒	☐	A description of any assumptions or corrections, such as tests of normality and adjustment for multiple comparisons
☐	☒	A full description of the statistical parameters including central tendency (e.g. means) or other basic estimates (e.g. regression coefficient) AND variation (e.g. standard deviation) or associated estimates of uncertainty (e.g. confidence intervals)
☒	☐	For null hypothesis testing, the test statistic (e.g. *F*, *t*, *r*) with confidence intervals, effect sizes, degrees of freedom and *P* value noted<br>*Give P values as exact values whenever suitable.*
☒	☐	For Bayesian analysis, information on the choice of priors and Markov chain Monte Carlo settings
☒	☐	For hierarchical and complex designs, identification of the appropriate level for tests and full reporting of outcomes
☒	☐	Estimates of effect sizes (e.g. Cohen's *d*, Pearson's *r*), indicating how they were calculated

Our web collection on statistics for biologists contains articles on many of the points above.

Software and code

Policy information about availability of computer code

Data collection	Beamline Scheduling Software on BL32XU at SPring-8
Data analysis	XDS, XSCALE, AIMELESS, PHENIX, COOT, PyMOL, and PRISM.

For manuscripts utilizing custom algorithms or software that are central to the research but not yet described in published literature, software must be made available to editors/reviewers. We strongly encourage code deposition in a community repository (e.g. GitHub). See the Nature Research guidelines for submitting code & software for further information.

Data

Policy information about availability of data

All manuscripts must include a data availability statement. This statement should provide the following information, where applicable:
- Accession codes, unique identifiers, or web links for publicly available datasets
- A list of figures that have associated raw data
- A description of any restrictions on data availability

Atomic structures have been deposited in the Protein Data Bank (PDB) with accession codes 6KRZ (AdipoR1 (A208)), 6KS0 (AdipoR1 (D208)), and 6KS1 (AdipoR2 (D219)).

Field-specific reporting

Please select the one below that is the best fit for your research. If you are not sure, read the appropriate sections before making your selection.

☒ Life sciences ☐ Behavioural & social sciences ☐ Ecological, evolutionary & environmental sciences

Life sciences study design

All studies must disclose on these points even when the disclosure is negative.

Sample size	N/A
Data exclusions	No data were excluded in this study.
Replication	All the experiments in the manuscript were reliably reproduced.
Randomization	N/A
Blinding	N/A

Reporting for specific materials, systems and methods

We require information from authors about some types of materials, experimental systems and methods used in many studies. Here, indicate whether each material, system or method listed is relevant to your study. If you are not sure if a list item applies to your research, read the appropriate section before selecting a response.

Materials & experimental systems

n/a	Involved in the study	
☐	☒	Antibodies
☐	☒	Eukaryotic cell lines
☒	☐	Palaeontology
☒	☐	Animals and other organisms
☒	☐	Human research participants
☒	☐	Clinical data

Methods

n/a	Involved in the study	
☒	☐	ChIP-seq
☒	☐	Flow cytometry
☒	☐	MRI-based neuroimaging

Antibodies

Antibodies used	Anti-FLAG M2 affinity agarose gel (#A2220) was purchase from SIGMA. The Fv fragment of an anti-AdipoR1/R2 antibody was produced by ourselves.
Validation	Anti-FLAG M2 antibody Affinity Gel (A2220 SIGMA) was used for the purification. The anti-AdipoR antibody produced by ourselves (DOI: 10.1007/s10969-014-9192-z) was used for the purification and crystallization, and the specificity was confirmed by the crystal structure.

Eukaryotic cell lines

Policy information about cell lines

Cell line source(s)	The cell lines Sf9, High Five, FreeStyle 293-F, and HEK293A were purchased from Invitrogen/Thermo Fisher Scientific, and their catalog numbers are 12659017, B85502, R79007, and R70507, respectively.
Authentication	The cell lines are supposed to have been authenticated by the supplyer (Invitrogen/Thermo Fisher Scientific).
Mycoplasma contamination	Sf9 cells, High Five cells, FreeStyle 293-F cells and HEK293A cells were not tested for mycoplasma contamination.
Commonly misidentified lines (See ICLAC register)	No commonly misidentified cell lines were used.
